# Supplementary material for: Competence of healthcare professionals in diagnosing and managing obstetric complications and conducting neonatal care: a clinical vignette-based assessment in district and subdistrict hospitals in northern Bangladesh
Source: BMJ Open. 2019 Aug 18;9(8):e028670. doi: 10.1136/bmjopen-2018-028670 (PMC6701613; doi:10.1136/bmjopen-2018-028670)
Supplement: Supplementary data [file bmjopen-2018-028670supp001.pdf]

S1 Appendix: Obstetric and neonatal vignette scenarios with scores for vignette action points

Case A (Eclampsia): Total score: 09
A 26-year-old woman who is 7 months pregnant comes in complaining of headaches, blurred vision and epigastric pain and her face looks swollen. In this facility, what would you usually do to establish a diagnosis?

1. Measure woman's blood pressure +*1 point if mentioned*
2. Check urine for protein +*1 point if mentioned*
3. Check her reflexes +*1 point if mentioned*
4. Check fetal heart rate +*1 point if mentioned*

Upon examination she had a blood pressure of 170/120, 3+ protein in her urine and brisk reflexes. How would she be managed at this facility?

1. Giving antihypertensive drug, e.g. hydralazine, labetalol, nifedipine +*1 point if mentioned*
2. Give Magnesium sulfate or, if not available, diazepam +*1 point if mentioned*
3. Give diuretics +*1 point if mentioned*
4. Have somebody stay with her all the time in case she starts having seizures +*1 point if mentioned*
5. Plan for delivery within the next 24 hours, or
6. Refer to other health facility immediately +*1 point if at least one out of two mentioned*

Case B (Antepartum haemorrhage): Total score: 10
A 35-year old woman who is 8 months pregnant comes to this facility because she has started to bleed heavily vaginally. She has no contractions and does not complain of any pain. In this facility, what would you usually do to establish a diagnosis?

1. Check woman's vital signs +*1 point if mentioned*
2. Check fetal heart rate +*1 point if mentioned*
3. Perform abdominal examination +*1 point if mentioned*
4. Will not perform vaginal examination +*1 point if mentioned*

The woman has a feeble pulse at 120/min, her systolic blood pressure is 85 and she is pale, sweating and breathing rapidly at 30 breaths per minute. Foetal heart sound is normal. There is no tenderness on abdominal examination. She is still bleeding vaginally, bright red blood. You suspect placenta praevia and therefore do not perform a vaginal examination. How would such a patient be managed now?

1. Elevate legs to increase return of blood to the heart +*1 point if mentioned*
2. Give IV fluids rapidly +*1 point if mentioned*
3. Give oxygen by mask or nasal cannula +*1 point if mentioned*
4. Do ultrasound to confirm diagnosis +*1 point if mentioned*
5. Prepare for Caesarian section or refer to hospital where caesarean section can be done +*1 point if mentioned*
6. Give blood transfusion +*1 point if mentioned*

Case C (Care for very low birth weight baby): Total score: 20
A 17-year-old woman pregnant for 8 months delivered a baby at home. The baby was weighed and found to be 1.4kg. The baby was referred to your facility and suppose you had admitted her for immediate care. What would you do for this baby?

1. Detain for thorough examination *1.50 point if mentioned*

2. Ensure breastfeeding is established and provide support if necessary *2.05 point if mentioned*
3. Put the baby in an incubator *2.13 point if mentioned*
4. Teach the mother to keep baby Skin-to-skin or KMC *1.92 point if mentioned*
5. Check cord dressing and other potential sources of infection *1.28 point if mentioned*
6. Encourage and ensure hygiene in care *1.12 point if mentioned*

Mother says the baby is not breastfeeding and was contemplating giving glucose solution. What would you do?

1. Watch her breastfeed her baby and teach her good positioning and attachment *3.03 point if mentioned*
2. Examine the baby's mouth to ensure there are no anatomical deformities *1.47 point if mentioned*
3. If baby not breast feeding, teach her to express the milk and feed with a clean cup *2.50 point if mentioned*
4. Encourage infant formula only if EBM is not possible and mother can afford *1.00 point if mentioned*
5. Educate her and encourage her to practice exclusive breastfeeding for 1st 6 months of the baby's life *2.00 point if mentioned*

Case D (Essential newborn care and resuscitation): Total score: 30
A woman presented here in labour. The FHR is more than 160bpm. On examination, her cervix was fully dilated, and the baby had the head in the perineum. The baby is delivered and is normal weight but did not cry after delivery; what would you do for this baby?

1. Dry quickly *score: 2.66 point if mentioned*
2. Examine and suction mouth *score: 2.16 point if mentioned*
3. Ensure extra warmth for the baby *score: 1.50 point if mentioned*
4. Use bag and mask to ventilate if baby does not cry after suctioning *score: 2.53 point if mentioned*
5. Apply cardiac massage if ventilation alone does not help *score: 1.16 point if mentioned*

Suppose the resuscitation was successful, what would you do next?

1. Initiate breastfeeding immediately *score: 3.31 point if mentioned*
2. skin-to-skin contact with mother *score: 4.34 point if mentioned*
3. Ensure and encourage hygiene *score: 2.34 point if mentioned*

During routine checking on the baby after about 2 hrs, you saw the baby sleeping alone and the mother is sleeping but not in touch with baby. There was no covering on the baby since it wriggled out of the mother's cloth. What would you do?

1. Feel if baby is too cold *score: 1.28 point if mentioned*
2. Take the temperature with a thermometer *score: 1.53 point if mentioned*
3. Give skin-to-skin care/kangaroo mother care by mother or put in incubator for rewarming *score: 3.94 point if mentioned*
4. Check the ward to see if windows are open and close them all as well as any fans on the ward to prevent draught *score: 1. 41 point if mentioned*
5. Let the mother breastfeed the baby immediately *score: 1.84 point if mentioned*
