# Supplementary material for: Competence of healthcare professionals in diagnosing and managing obstetric complications and conducting neonatal care: a clinical vignette-based assessment in district and subdistrict hospitals in northern Bangladesh
Source: BMJ Open. 2019 Aug 18;9(8):e028670. doi: 10.1136/bmjopen-2018-028670 (PMC6701613; doi:10.1136/bmjopen-2018-028670)
Supplement: Author's manuscript [file bmjopen-2018-028670.draft_revisions.pdf]

BMJ Open is committed to open peer review. As part of this commitment we make the peer review history of every article we publish publicly available.

When an article is published we post the peer reviewers' comments and the authors' responses online. We also post the versions of the paper that were used during peer review. These are the versions that the peer review comments apply to.

The versions of the paper that follow are the versions that were submitted during the peer review process. They are not the versions of record or the final published versions. They should not be cited or distributed as the published version of this manuscript.

BMJ Open is an open access journal and the full, final, typeset and author-corrected version of record of the manuscript is available on our site with no access controls, subscription charges or pay-per-view fees (http://bmjopen.bmj.com).

If you have any questions on BMJ Open's open peer review process please email info.bmjopen@bmj.com

Competence of the Health Care Provider in Diagnosing and Managing Obstetric Complications and Conducting Neonatal Care: A Clinical Vignette Based Assessment in District and Sub-District Hospitals in Bangladesh

Journal:	*BMJ Open*
Manuscript ID	bmjopen-2018-028670
Article Type:	Research
Date Submitted by the Author:	24-Dec-2018

Complete List of Authors:	Khan, Abdullah Nurus Salam; University of South Carolina, Health promotion, education and behavior; International Centre for Diarrhoeal Disease Research Bangladesh, Maternal and Child Health Division<br>Karim, Farhana; International Centre for Diarrhoeal Disease Research Bangladesh, Maternal and Child Health Division<br>Chowdhury, Mohiuddin Ahsanul Kabir; University of South Carolina; International Centre for Diarrhoeal Disease Research Bangladesh, Maternal and Child Health Division<br>Zaka, Nabila; UNICEF USA,<br>Manu, Alexander; Liverpool School of Tropical Medicine<br>Arifeen, Shams E.; ICDDR B, Maternal and Child Health Division (MCHD)<br>Billah, Sk; International Centre for Diarrhoeal Disease Research, Maternal and Child Health Division

Keywords:	Human resource management < HEALTH SERVICES ADMINISTRATION & MANAGEMENT, Quality in health care < HEALTH SERVICES ADMINISTRATION & MANAGEMENT, Organisation of health services < HEALTH SERVICES ADMINISTRATION & MANAGEMENT, OBSTETRICS, PERINATOLOGY, PUBLIC HEALTH

SCHOLARONE™ Manuscripts

Title:

Competence of the Health Care Provider in Diagnosing and Managing Obstetric Complications and Conducting Neonatal Care: A Clinical Vignette Based Assessment in District and Sub-District Hospitals in Bangladesh

Authors:

Abdullah Nurus Salam Khan[1,2], Farhana Karim[1], Mohiuddin Ahsanul Kabir Chowdhury[1,2], Nabila Zaka[3], Alexander Manu[4], Shams El Arifeen[1], Sk. Masum Billah[1]

[1] Maternal and child health division, International Centre for Diarrhoeal Disease Research, Bangladesh (icddr,b), Dhaka, Bangladesh

[2] Arnold School of Public Health, University of South Carolina

[3] Health Section, Maternal and Newborn Health, UNICEF, New York, USA

[4] Centre for Maternal and Newborn Health, Liverpool School of Tropical Medicine, Liverpool, UK

# Corresponding author

Name: Sk Masum Billah

Address: Maternal and Child Health Division, International Centre for Diarrhoeal Disease Research, Bangladesh (icddr,b);

68 Shahid Tajuddin Ahmed Sarani, Mohakhali, Dhaka 1212, Bangladesh

Telephone: +880 171 7416117

Fax: +880 298 27077

Email: billah@icddrb.org

For peer review only

For peer review only - http://bmjopen.bmj.com/site/about/guidelines.xhtml

Abstract:

Background: This study assesses the competency of maternal and neonatal health (MNH) providers of government health facilities in Bangladesh using clinical vignettes. The study also measures whether the provider's characteristics and provision of related services in their health facilities influence their competencies.

Methods: A total of 134 MNH care providers in 15 government hospitals were interviewed during August and September 2016 using clinical vignettes on obstetric complications (antepartum haemorrhage and pre-eclampsia) and neonatal care (low-birthweight and immediate newborn care), to assess their competency. Median vignette scores were compared across different categories of provider's characteristics and facility's MNH service provision to measure association of competency with these variables. Kruskal-Wallis test was performed to identify the significance of association considering p-value<0.05 as statistically significant.

Results: The competency of MNH providers was overall poor; About 10% and 24% of the providers received 'high' score (>75% of total) in maternal and neonatal vignettes, respectively. Medical doctors had higher competency than nurses and midwives (score=11 vs. 8 out of 19, respectively; p=0.0002) for maternal vignettes, but similar competency for neonatal vignettes (score=30.3 vs. 30.9 out of 50, respectively). Providers working in health facilities with higher utilization of normal deliveries, had better competency than their counterparts. Providers had higher competency in newborn vignettes (significant) and maternal vignettes (statistically not significant) if they worked in health facilities that provided more specialized newborn care services and emergency obstetric care, respectively, in last 6 months.

Conclusions: Despite the overall poor competency of MNH providers working in health facilities, exposure to higher number of cases at workplace was found associated with provider's competence. Arrangement of periodic skill-and-drill based in-service training for MNH providers especially the nurses and midwives in high-utilization neighbouring health facilities could be a feasible intervention to improve their knowledge and skill in obstetric and neonatal care.

For peer review only - http://bmjopen.bmj.com/site/about/guidelines.xhtml

Strengths and limitations:

- The study assesses the competence (knowledge and skill) of doctors, nurses and midwives in managing complicated obstetric and neonatal cases using clinical vignettes
- The study identifies the association of provider level factors such as provider cadre, work experience, training, and health facility level factors such as facility types, service provision, obstetric caseload with the competence of providers
- The study provides guidance on improving the competency of health care providers working at health facilities in low-resource settings
- The study does not validate the competency assessed by clinical vignettes by comparing it with observation of or chart abstraction of performed services at clinical settings

For peer review only

For peer review only - http://bmjopen.bmj.com/site/about/guidelines.xhtml

Background:

Maternal and neonatal survival has received significant priorities in the global agenda of Millennium Development Goals (MDG), and subsequently in Sustainable Development Goals (SDG) [1-3]. Since the adoption of MDGs, the maternal mortality ratio (MMR) and the neonatal mortality rate (NMR) have been reduced by 44% and 49% between 1990 and 2015, respectively [4, 5]. Despite the progress achieved in reducing MMR and NMR, 303,000 mothers and 4 million newborns are still estimated to be dying each year worldwide and 99% of them are dying in developing regions [4, 5]. A WHO systematic review shows around 27% dies of haemorrhage and 14% dies due to complications arising from hypertensive disorders during pregnancy including preeclampsia [6]. For newborns, the major reasons for mortality are preterm birth, birth asphyxia and infections [7, 8]. The evidence indicates that the highest mortality risk for both mothers and their babies is on the first day of birth [9]. It is estimated that 16 to 33% of maternal mortality can be averted if the major causes of death can be managed timely and efficiently by skilled attendance during childbirth [10]. Although, the mere presence of skilled birth attendant in health facilities equipped with key signal functions of emergency obstetric care does not ensure standard practices of intrapartum and immediate postpartum care and subsequently fail to improve survival of mothers and newborns [11]. In addition to the midwifery skills, a skilled birth attendant should be able to diagnose and manage complications arising at the time of childbirth and refer the cases which are beyond their competence or not possible to manage in a particular setting [12].

The quality of care gap with skilled birth attendance is crucial for low and middle-income countries (LMIC) as they are struggling to increase institutional delivery with limited skilled workforce and resources to prevent their major share of global maternal and neonatal deaths [13]. With an aim to improve the overall maternal and neonatal health, LMICs often have to identify and prioritize the scopes of resources allocation for improving the competencies of health care providers in maternity and neonatal wards in facilities [14]. A quick and comprehensive way of assessing the competence of health care providers is to use clinical vignettes or written case simulations. This is an inexpensive and validated way to reflect on performance and quality of health workers' actual practice [15, 16] especially in low-income settings where chart documentation is often incomplete for obstetric cases [17, 18].

For peer review only - http://bmjopen.bmj.com/site/about/guidelines.xhtml

This paper aims to assess the competency of managing maternal and newborn complications among health care providers in district and sub-district level health facilities of Bangladesh using clinical vignettes. The paper also explores whether individual-level factors such as provider type, training status, work experience and facility-level factors such as facility type, obstetric caseloads i.e. annual delivery conducted in health facilities, readiness for emergency obstetric care are associated with the competence of the care providers. Findings from this paper will be useful to bring procedural changes in health facilities to improve overall health outcome and increase the accessibility to skilled delivery services in health facilities in LMICs.

Methodology:

Study design and study sites:

This study draws on the findings from a cross-sectional assessment conducted for the baseline evaluation of "Every Mother Every Newborn Quality Improvement (EMEN QI)" initiatives, jointly implemented by Government of Bangladesh, UNICEF headquarter and UNICEF Bangladesh. The initiative focuses on implementing quality of care standards for maternal and neonatal health care delivery in the selected health facilities of three neighbouring districts– Kurigram, Lalmonirhat and Gaibandha, situated in the northern part of Bangladesh. From each of the districts, four sub-district health facilities or Upazila Health Complex (UHC) and one district hospital (DH) were selected. All UHCs are primary level referral facilities designated to provide at least basic emergency obstetric and newborn care (BEmONC). The DHs, secondary level health facilities, offer comprehensive emergency obstetric and newborn care (CEmONC) services. The data were collected between August and September 2016.

Selection of MNH care providers:

From 3 DHs and 12 UHCs, a total of 134 health care providers who were posted and present at these facilities on the assessment day and were responsible to conduct normal deliveries, manage obstetric or neonatal emergencies, were selected for the assessment. The 134 respondents included 39 medical doctors, 91 nurses and 4 midwives.

Administration of vignettes:

For peer review only - http://bmjopen.bmj.com/site/about/guidelines.xhtml

We used two vignettes (Case A and B) to describe obstetric complications and two vignettes (Case C and D) on newborn care. Case A and Case B describe scenarios on pregnant women with signs of preeclampsia and antepartum haemorrhage, respectively, and both cases include separate sections on 'diagnosis' and 'management' of those conditions. Case C describes a baby with foetal distress and comprises three parts- i) resuscitation ii) immediate newborn care, and iii) thermal care. Case D is about very low birth weight baby and comprises of two parts – i) immediate management, and ii) counselling caregivers on breastfeeding.

A team of trained research physicians administered all four clinical vignettes among the health care providers through face-to-face interview using a structured questionnaire. At first, the research physician read out the scenarios to the provider and asked for specific actions to be taken for each of the scenarios. All responses were matched with a pre-defined list of vignette action points (Appendix S1) and were considered as correct if they matched with the listed action points. The research physicians were trained on the vignette scenarios and the definition of each vignette action points. Since all four vignette scenarios were read out and responses were collected for each, there was no incomplete data. Intra- and inter-observer variability in measuring the responses were less likely to occur as the responses were structured.

Assessing the provision of MNH services:

In 15 health facilities, a separate team of research assistants extracted the information on the number of conducted deliveries and the provision of emergency obstetric care and specialized newborn services in the last 6 months from health facility records using a checklist. Emergency obstetric care included administration of injectable antibiotics, injectable oxytocin, injectable anticonvulsant; manual removal of retained placenta, retained products of conception, and provision of assisted vaginal delivery, blood transfusion and caesarean delivery. Specialized newborn services were the provision of kangaroo mother care (KMC), basic resuscitation (clearing of airway, stimulation, bag and mask ventilation), advanced resuscitation (intubation, chest compression), administration of oxygen, intravenous fluid, and antenatal corticosteroid.

Data analysis:

To analyze the total vignette score obtained by the respondents, each of the 'actions' was assigned to either equal score (Case A and B) or weighted ones (Case C and D). The detailed

For peer review only - http://bmjopen.bmj.com/site/about/guidelines.xhtml

methodology of assigning the scores are mentioned elsewhere [19, 20] and the authors performed adequate sensitivity analysis to choose between equal or weighted scoring.

Based on the assigned scores, summative scores were calculated for the vignette cases on maternal and neonatal complication. Median vignette scores were compared between different categories of health care providers and their working health facilities using Kruskal-Wallis tests. Providers were categorized based on their cadres, total working experience, training status, and working station in the facility; and health facilities were categorized based on their level of care, annual case-load of delivery and preparedness for emergency management of maternal and neonatal complication. Facility-level readiness was reported using frequency and percentage of the readiness indicators. The indicators were considered 'available' only if the activities related to these indicators was performed in the health facility within six months prior to the assessment. P-value less than 0.05 was considered as statistically significant for Kruskal-Wallis test. The analysis was performed using Stata 13.0.

Patient and public involvement:

The research questions and outcome measures were related to assessing the knowledge and skill of government health care provider's in managing obstetric and neonatal complications using clinical vignettes. In selection of cases for clinical vignettes, the critical cases were chosen and presented as they would occur among pregnant women and their newborn in the context of Bangladesh. Patients or pregnant women were not involved in the design of, in the recruitment to and conduct of the study. The results will be disseminated among the health managers and MNH providers in the government hospitals where the study was conducted.

Ethical approval:

The Ethical Review Committee (ERC) of icddr,b approved the study (protocol number 16024). Informed written consent was sought from the Health care providers and health facility managers before conducting interview.

Result:

Delivery utilization and provision of emergency obstetric and specialized neonatal care:

For peer review only - http://bmjopen.bmj.com/site/about/guidelines.xhtml

For peer review only

Table 1 presents the status of delivery utilization and readiness of the surveyed health facilities to provide emergency obstetric and newborn care by facility levels- district and sub-district. During preceding three years since the assessment, average number of deliveries conducted in a year was less than 600 (low-utilization) in 10 out of 12 sub-district hospitals, whereas in two out of three district hospitals and one sub-district hospital the average number was more than 900 a year (high-utilization) and rest two of the 15 health facilities were between 600-900 a year (medium-utilization). All signal functions of comprehensive emergency obstetric care (CEmOC) but assisted vaginal delivery were performed in all three district hospitals within 6 months prior to the assessment. However, provision of injectable anticonvulsant, blood transfusion and caesarean delivery were performed in 42%, 25% and 8% of the 12 sub-district hospitals, respectively. Provision of Kangaroo Mother Care (KMC) was not practised in any of the facilities. For birth asphyxia management, basic resuscitation procedures such as airway clearing, stimulation and bag-mask ventilation were practised in all the health facilities within 6 months prior to the assessment, but advanced resuscitation measures such as intubation or chest compression were only conducted in two district- and two sub-district hospitals within the same time period.

Table 1: Annual delivery utilization, and provision of emergency obstetric and specialized neonatal care in health facilities within 6 months prior to the survey

**Indicators**	**Total, n=15**	**District hospital, n=3**	**Sub-district hospital, n=12**
**Annual delivery conducted on average**			
Low; <600	10 (66.7)	0 (0.0)	10 (83.3)
Medium; 600-900	2 (13.3)	1 (33.3)	1 (8.3)
High; >900	3 (20.0)	2 (66.7)	1 (8.3)
**Provision of emergency obstetric care**			
Injectable antibiotics administration	15 (100.0)	3 (100.0)	12 (100.0)
Injectable oxytocin administration	15 (100.0)	3 (100.0)	12 (100.0)
Injectable anticonvulsant administration	8 (53.3)	3 (100.0)	5 (41.7)
Manual removal of retained placenta	14 (93.3)	3 (100.0)	11 (91.7)

For peer review only - http://bmjopen.bmj.com/site/about/guidelines.xhtml

Manual removal of retained products of conception	14 (93.3)	3 (100.0)	11 (91.7)
Assisted vaginal delivery	0 (0.0)	0 (0.0)	0 (0.0)
Blood transfusion services	6 (40.0)	3 (100.0)	3 (25.0)
Caesarean delivery	4 (26.7)	3 (100.0)	1 (8.3)
**Provision of specialized care for newborn**			
Provision of Kangaroo Mother Care (KMC)	0 (0.0)	0 (0.0)	0 (0.0)
Basic resuscitation[1]	15 (100.0)	3 (100.0)	12 (100.0)
Advanced resuscitation[2]	4 (26.7)	2 (66.7)	2 (16.7)
Oxygen administration	15 (100.0)	3 (100.0)	12 (100.0)
IV fluid administration	14 (93.3)	3 (100.0)	11 (91.7)
Antenatal corticosteroid (ACS) administration	3 (20.0)	2 (66.7)	1 (8.3)

[1] Clearing of airway, stimulation, bag and mask ventilation

[2] Intubation, chest compression

Characteristics of health care providers:

The mean age of 134 health care providers was 38 years, however, the mean age of nurses and midwives was higher than the medical doctors (40 vs. 32 years) (table 2). About one-third of doctors and all the nurses and midwives were female. Among the interviewed providers, 84% and 62% were working in maternity and neonatal wards, respectively. However, for half of the providers both working areas overlapped as they had experience of working in both wards. None of the medical doctors had more than five years of working experience whereas 59% of the nurses and midwives had so. A higher proportion of nurses and midwives had ever received any in-service training on obstetric complications than the doctors (96% vs. 44%) and about 45% of all providers had ever received any in-service training on newborn complications. Within 12 months prior to the assessment, only 7% and 29% providers received any training on obstetric and newborn complications, respectively.

Table 2: Characteristics of health care providers

Characteristics	Total, n=134	Medical doctor, n=39	Nurse and midwife, n=95
Mean age	37.7±7.5	32.2±6.1	40.0±6.9
Female providers	79.9	30.8	100.0
**Type of health facility where posted**			

For peer review only - http://bmjopen.bmj.com/site/about/guidelines.xhtml

District hospital	24.6	17.9	27.4
Sub-district hospital	75.4	82.1	72.6
**Majorly works in**			
Both maternity and neonatal wards	51.5	56.4	49.5
Maternity ward	83.6	87.2	82.1
Neonatal ward	61.9	61.5	62.1
**Work experience**			
Less than 1 year	8.2	17.9	4.2
1-5 years	50.0	82.1	36.8
More than 5 years	41.8	0.0	58.9
**Ever received in-service training on**			
Management of obstetric complication	80.6	43.6	95.8
Management of newborn complication	44.8	51.3	42.1
**Received training in the last 12 months on**			
Management of obstetric complication	6.7	5.1	7.4
Management of newborn complication	29.1	28.2	29.5

Competence of providers in diagnosing and managing maternal and neonatal complication:

For vignettes dealing with obstetric complications (case A and B), the median summative score was 8 with interquartile range (IQR) of 6 to 11 (table 3). About 60% of the providers received 'low' score i.e. less than 50% of the maximum possible score and only 10% achieved 'high' score (more than 75% of the total score). Between the two obstetric cases, providers had slightly better knowledge about diagnosis and management of eclampsia (case A) than antepartum haemorrhage (case B) (49% received 'moderate' to 'high' score vs. 38%, respectively). Moreover, 51% of the provider had 'moderate' to 'high' score regarding the diagnosis of APH and Eclampsia in comparison to 36% who had the similar competency of knowledge regarding management of these cases.

The median summative vignette score for neonatal complications (case C and D) was 30.8 (IQR: 23.9, 36.6) where 50 was the maximum possible score. About 70% of the providers could achieve at least 50% of the total score in newborn vignettes. Between the two cases, providers' competence on provision of essential newborn care (case D) was better than that for management of very low birth weight baby (case C) as evident from the categorical classifications of scores (78% received 'moderate' to 'high' score vs. 58%, respectively).

Table 3: Median and categorical classification of vignette scores

Vignette Cases	Total range	Median score (IQR)	Categorical score (%), N=134		
			High	Moderate	Low

For peer review only

For peer review only - http://bmjopen.bmj.com/site/about/guidelines.xhtml

			(>75%)	(50-75%)	(<50%)
**Maternal complication**					
Case A (Eclampsia)	0-09	4 (3,6)	12.7	36.6	50.7
Case B (Antepartum hemorrhage)	0-10	4 (2,6)	8.2	29.9	61.9
Case A+B (Diagnosis component)	0-09	5 (3,6)	11.2	40.3	48.5
Case A+B (Management component)	0-10	4 (2,6)	8.2	27.6	64.2
Case A+B (Total)	0-19	8 (6,11)	10.4	29.9	59.7
**Neonatal complication**					
Case C (Care for VLBW babies)	0-20	11.1 (8.4, 14.9)	23.9	34.3	41.8
Case D (Essential newborn care)	0-30	19.8 (15.6,24.4)	30.6	47.0	22.4
Case C+D (Total)	0-50	30.8 (23.9, 36.6)	23.9	46.3	29.9

IQR= Interquartile range; VLBW= Very low birth weight

Table 4 presents the distribution of median total vignette scores for maternal and neonatal complications across different attributes of the providers and their working health facilities. For maternal complications, the median scores differed significantly between the types of providers (p=0.0002) with medical doctors achieving a higher score than nurses or midwives. Neither of the total vignette scores was significantly different among the providers who worked in both maternal and neonatal wards or only in one of these wards or between levels of health facilities where they were posted. Providers' duration of work experience or their receipt of training on management of obstetric or neonatal complication also did not make any significant difference in their competencies. However, providers working in high utilization health facilities with more than 900 deliveries annually had a better competency in both maternal and neonatal complications management (p=0.0005 and p=0.004, respectively). Providers working in health facilities which provided more than 5 out of 7 EmOC services had statistically non-significant (p=0.6819) but higher competency in maternal vignettes. Providers had significantly higher competency in newborn vignettes (p=0.0361) if they were working in health facilities which were better equipped to provide emergency management for newborn complications.

Table 4: Median scores of maternal and neonatal complication vignettes within different categories of health care providers

**Characteristics**	**Maternal complication (Case A and B)**		**Neonatal complication (Case C and D)**	
	**Median score[1] (IQR)**	**p-value[2]**	**Median score[1] (IQR)**	**p-value[2]**
**Type of provider**				
Medical doctor	11 (7, 14)	**0.0002**	30.3 (21.0, 45.3)	0.6682

For peer review only - http://bmjopen.bmj.com/site/about/guidelines.xhtml

Nurse and midwives	8 (6, 10)		30.9 (25.5, 35.5)	
**Majorly works in**				
Both maternity and neonatal ward	9 (6, 11)	0.7540	30.9 (24.1, 36.0)	0.3636
Maternity ward only	8 (6, 11)		27.9 (23.0, 36.6)	
Neonatal ward only	8 (7, 13)		32.3 (26.0, 39.2)	
**Work experience**				
Less than 1 year	10 (7, 12)	0.1099	30.9 (23.3, 37.5)	0.8443
1-5 years	9 (7, 12)		31.6 (22.1, 39.2)	
More than 5 years	7.5 (5, 10)		29.7 (25.7, 35.3)	
**Ever trained on management of obstetric complication**				
Yes	8 (6, 11)	0.0931	30.9 (24.5, 36.1)	0.6628
No	9 (7, 13)		29.5 (20.5, 45.3)	
**Ever trained on management of neonatal complication**				
Yes	8.5 (5, 11)	0.8005	30.8 (21.8, 36.3)	0.3817
No	8 (6, 12)		30.8 (25.9, 37.6)	
**Received training in last 12 months on the management of obstetric complication**				
Yes	10 (6, 12)	0.5456	30.3 (27.6, 36.3)	0.3789
No	8 (6, 11)		30.7 (23.3, 36.6)	
**Received training in last 12 months on the management of neonatal complication**				
Yes	9 (6, 12)	0.3674	31.9 (23.0, 37.5)	0.5567
No	8 (6, 11)		30.1 (24.3, 36.6)	
**Type of health facility where posted**				
District hospital	8 (7, 11)	0.6682	28.7 (25.5, 35.5)	0.4618
Sub-district hospital	8 (6, 11)		31.3 (23.3, 37.7)	
**Annual delivery conducted in the health facility**				
Low; <600	8 (6, 11)	**0.0005**	30.0 (22.9, 36.3)	**0.004**
Medium; 600-900	7 (5, 9)		27.4 (23.3, 34.3)	
High; >900	12 (9, 15)		34.5 (30.2, 46.1)	
**Provision of EmOC[3]**				
5 or less services	8 (6, 12)	0.6819	31.3 (23.2, 38.2)	0.4164
More than 5	9 (7, 11)		29.1 (25.5, 35.5)	
**Provision of emergency management for neonatal complication[4]**				
3 or less services	8 (6, 11)	0.2722	29.3 (23.0, 35.7)	**0.0361**
More than 3	8 (7, 12.5)		31.8 (26.0, 38.7)	

[1] Score range for maternal complication is 0-19 and for neonatal complication, the range is 0-50

[2] p-value is obtained from the Kruskal-Wallis test

For peer review only

[3] Provision of injectable antibiotic, oxytocin, and anticonvulsant; Manual removal of placenta and retained products, performing blood transfusion and caesarean-sections within 6 months prior to survey

[4] Provision of Kangaroo Mother Care (KMC), basic and advanced resuscitation, administration of oxygen, intravenous fluids, and antenatal corticosteroids within 6 months prior to survey

Competency by provider cadres in low, medium and high utilization health facilities:

Figure 1 and Figure 2 show the vignette scores for obstetric and neonatal complications, respectively, across provider cadres by health facility's annual delivery utilization. It is observed that the competence of the providers working in low utilization facilities fall majorly in 'low' to 'moderate' categories and the median scores do not differ significantly between medical doctors and nurses or midwives. However, medical doctors working in high utilization facilities shows 'high' competency in comparison to most nurses or midwives, especially in newborn complication management.

Figure 1: The vignette scores for obstetric complications by providers cadre and type of facility

Figure 2: The vignette scores for neonatal complications by providers cadre and type of facility

Discussion:

The study finds poor competency of diagnosing and managing critical obstetric and neonatal conditions among the health care providers of selected district and sub-district hospitals in Bangladesh. The deficient practice of managing obstetric emergencies or providing specialized newborn care along with very low coverage of recent refresher training might have reflected in poor competencies of these providers. The competency, as measured by the vignette scores, varied significantly among the type of providers and was associated with obstetric caseloads of the health facilities.

All health care providers were working in obstetric or neonatal wards in the selected facilities and were expected to achieve standard competencies for the first line management of the scenarios presented in both the maternal and the newborn vignettes [21]. The vignette score indicates that majority of the providers scored less than 50% of the total i.e. 'low' vignette scores for the obstetric complications- antepartum haemorrhage (APH) and eclampsia but showed better

For peer review only - http://bmjopen.bmj.com/site/about/guidelines.xhtml

competencies for newborn complications. This could result from the poor retention of knowledge of managing obstetric complication due to lack of recent training. Data shows that providers were less likely to receive any training on management of maternal complication within the last 12 months than neonatal complications (7% vs. 30%) whereas the majority of them had ever received in-service training on these topics. Although capacity development through targeted training of the maternal, newborn and child health (MNCH) care providers is identified as a critical intervention to improve health outcome [22], this often fails to meet the desired outcome due to inadequate post-training monitoring, follow-up and periodic refresher training [23]. The study also identified that both vignette scores were higher among the providers who work in health facilities with 'high' caseload of conducting deliveries but remained nearly similar in 'low' and 'medium' utilization facilities. This association underscores the fact that experiential learning or on-the-job experiences can improve the competence from what they have learnt through academic training alone [24, 25]. The comprehensive package of training for emergency obstetric and newborn care (EmONC) could therefore be adapted to include skill-and-drill based in-service training in 'obstetric skills laboratories' or simulation centres within or nearby the health facility premises [26]. Operations researches within government's existing EmONC training programme should further explore for efficient ways to incorporate such interventions in low-resource settings.

Our study also identifies that the doctors had significantly better knowledge score than nurses and midwives in maternal complication vignettes but similar score in newborn vignettes. With the higher extent of academic and clinical training, it is likely that doctors would have higher competency score than nurses and midwives in the maternal vignettes which includes more intensive clinical procedures than the newborn scenarios. But their similar performance in newborn vignettes indicates that more advanced pre-service clinical training of the providers does not always translate into better knowledge and skill, and similar evidence was found for sick child management in a multi-country study [27]. This directs the policymakers to consider the role of task-shifting from doctors to nurses and midwives to maximize the productive efficiency in health facilities of LMICs particularly for low intensive procedures like immediate newborn care or identification of obstetric complications and referral [28, 29]. As in Bangladesh, the newly deployed midwifery cadre is expected to work with their full capacity within the health system according to the Midwifery Act [30].

For peer review only - http://bmjopen.bmj.com/site/about/guidelines.xhtml

The study found that median maternal vignette scores were higher, although statistically not significant, among the providers who worked in facilities that were better equipped to provide emergency obstetric services whereas for newborn vignettes the scores were significantly higher in facilities that could provide better newborn specialized services. This indicates that poor preparedness of the facilities could result in the poor competency of the providers who are designated to manage maternal and newborn complications through infrequent exposure to such critical cases. In the assessed sub-district hospitals, provision of blood transfusion which is required in case of severe APH or provision of injectable magnesium sulphate for early stabilization of pre-eclampsia were infrequent in the last six months. The gap was also profound for conducting caesarean sections, advanced resuscitation for newborn and antenatal corticosteroid administration. Kangaroo Mother Care (KMC), an evidence-based care for low birth weight babies, was not provided in any of the assessed health facilities. The literature suggests that the competence of providers is shaped from actively engaging in evidence-based practices [25] and to implement such practices, the readiness of the health facilities should be optimum for the providers to carry out the intended procedures [31, 32]. Improving the competence of health care provider to ensure the procedural quality of care should, therefore, require structural improvement of the health facilities to allow them to conduct the required tasks.

The findings of the study should be cautiously interpreted as the study assesses the competence using clinical vignettes which explores what one should do at an expected level, not what one actually performs in real settings [33]. Although in absence of proper chart documentation or lack of opportunity to directly observe providers managing complicated obstetric cases clinical vignette provides a cheap alternative to assess both knowledge and skill of providers [16]. The study was only conducted in the district and sub-district level health facilities excluding the first level health facilities where childbirth by normal delivery takes place [21]. However, the first level facilities in Bangladesh are not equipped or entitled to provide EmONC services and monthly utilization of normal delivery is very low [21].

Conclusion:

For peer review only - http://bmjopen.bmj.com/site/about/guidelines.xhtml

Clinical vignettes could be a useful tool to assess the competency, a key predictor of performance, of MNCH care providers in low resource settings. Provider cadre and obstetric case load of the facilities were associated with the competence of health care providers. The arrangement of periodic skill-and-drill based in-service and refreshers training for MNCH providers using high-utilization neighbouring health facilities as ‘obstetric skills laboratory’ could be a feasible intervention to improve the competence of the MNH providers in LMICs.

Author’s Contribution

SEA, SMB, NZ and MAKC designed the study and SMB, MAKC, and ANSK conceptualized the paper. ANSK, FK, and MAKC were involved in data acquisition and literature review. ANSK and FK conducted the analysis of paper and NZ, AM, and SEA guided additional analysis. ANSK and FK prepared the first draft. MAKC, NZ, AM, SEA and SMB contributed in revision and preparation of the final draft. All authors have reviewed and approved the final manuscript.

Competing interests

No conflict of interest is declared by any author.

Funding

The study was conducted with funding support from Bill and Melinda Gates Foundation for EMEN Quality Improvement Initiative to UNICEF Headquarter (Bill and Melinda Gates Foundation Grant number OPP1112117, UNICEF and BMGF partnership Grant).

Availability of data and material

The data that support the findings of this study are available from the corresponding author upon reasonable request.

Acknowledgement

We acknowledge the support received from Quality Improvement Secretariat of Ministry of Health and Family Welfare and UNICEF Bangladesh to facilitate data collection. We are grateful

For peer review only - http://bmjopen.bmj.com/site/about/guidelines.xhtml

to the research participants i.e. the health care providers, and hospital managers for allowing us to conduct the study. We are also thankful to the data collection and entry team for their commitment and sincere effort.

For peer review only

For peer review only - http://bmjopen.bmj.com/site/about/guidelines.xhtml

References:

1. Sachs, J.D., *From millennium development goals to sustainable development goals.* The Lancet, 2012. **379**(9832): p. 2206-2211.
2. WHO, U. and C. Mathers, *Global strategy for women's, children's and adolescents' health (2016-2030).* Organization, 2017. **2016**(9).
3. United Nations. *Sustainable development goals: 17 goals to transform our world*. 2015; Available from: https://www.un.org/sustainabledevelopment/sustainable-development-goals/.
4. World Health Organization, *Trends in maternal mortality: 1990 to 2015 Estimates by WHO, UNICEF, UNFPA, World Bank Group and the United Nations Population Division*. 2015: Geneva, Switzerland.
5. United Nations Inter-agency Group for Child Mortality Estimation (UN IGME). *Levels & trends in child mortality: Report 2017, Estimates developed by the UN Inter-agency Group for Child Mortality Estimation*. 2017: United Nations Children's Fund, New York.
6. Say, L., et al., *Global causes of maternal death: a WHO systematic analysis.* Lancet Glob Health, 2014. **2**(6): p. e323-e333.
7. Liu, L., Johnson, H. L., Cousens, S., Perin, J., Scott, S., Lawn, J. E., & Black, R. E. , 379, *Global, regional, and national causes of child mortality: an updated systematic analysis for 2010 with time trends since 2000.* Lancet, 2012. **379**(9832): p. 2151-2161.
8. Lawn, J.E., et al., *4 million neonatal deaths: when? Where? Why?* Lancet, 2005. **365**(9462): p. 891-900.
9. Ronsmans, C., W.J. Graham, and Lancet Maternal Survival Series steering group., *Maternal mortality: who, when, where, and why.* Lancet, 2006. **368**(9542): p. 1189-1200.
10. Graham WJ, B.J., Bullough CHW, *Can skilled attendance at delivery reduce maternal mortality in developing countries?* Safe Motherhood strategies: a review of the evidence 2001. **17**: p. 97-130.
11. Souza, J.P., et al., *Moving beyond essential interventions for reduction of maternal mortality (the WHO Multicountry Survey on Maternal and Newborn Health): a cross-sectional study.* Lancet, 2013. **381**(9879): p. 1747-1755.

For peer review only - http://bmjopen.bmj.com/site/about/guidelines.xhtml

12. World Health Organization., *UNFPA/UNICEF/World Bank Statement: Reduction of maternal mortality.* World Health Organization, 1999.
13. Freedman, L.P., et al., *Transforming health systems to improve the lives of women and children.* Lancet, 2005. **365**(9463): p. 997-1000.
14. Das, J.K., et al., *Evidence from facility level inputs to improve quality of care for maternal and newborn health: interventions and findings.* Reprod Health, 2014. **11**(2): p. S4.
15. Peabody, J.W., et al., *Comparison of vignettes, standardized patients, and chart abstraction: a prospective validation study of 3 methods for measuring quality.* Jama, 2000. **283**(13): p. 1715-1722.
16. Peabody, J.W., et al., *Measuring the quality of physician practice by using clinical vignettes: a prospective validation study.* Annals of internal medicine, 2004. **141**(10): p. 771-780.
17. Landry, E., et al., *Assessing the quality of record keeping for cesarean deliveries: results from a multicenter retrospective record review in five low-income countries.* BMC Pregnancy Childbirth, 2014. **14**(1): p. 139.
18. Wilunda, C., et al., *Availability, utilisation and quality of maternal and neonatal health care services in Karamoja region, Uganda: a health facility-based survey.* Reprod Health, 2015. **12**(1): p. 30.
19. Vesel, L., et al., *Quality of newborn care: a health facility assessment in rural Ghana using survey, vignette and surveillance data.* BMJ Open, 2013. **3**(5): p. e002326.
20. Lohela, T.J., et al., *Competence of health workers in emergency obstetric care: an assessment using clinical vignettes in Brong Ahafo region, Ghana.* BMJ Open, 2016. **6**(6): p. e010963.
21. Ministry of Health and Family Welfare (MOHFW), *Bangladesh Health Bulletin 2017.* 2018, Management Information System: Directorate General of Health Services: Bangladesh.
22. Nyamtema, A.S., D.P. Urassa, and J. van Roosmalen, *Maternal health interventions in resource limited countries: a systematic review of packages, impacts and factors for change.* BMC Pregnancy Childbirth, 2011. **11**(1): p. 30.
23. Islam, M.T., et al., *Implementation of emergency obstetric care training in Bangladesh: lessons learned.* Reprod Health Matters, 2006. **14**(27): p. 61-72.

For peer review only - http://bmjopen.bmj.com/site/about/guidelines.xhtml

24. Spencer, L.M., *Competency assessment methods.* What Works: Assessment, Development, and Measurement, 1997. **1**: p. 1-36.

25. Kak, N., B. Burkhalter, and M.-A. Cooper, *Measuring the competence of healthcare providers.* Operations Research Issue Paper, 2001. **2**(1): p. 1-28.

26. Utz, B., T. Kana, and N. Van Den Broek, *Practical aspects of setting up obstetric skills laboratories–a literature review and proposed model.* Midwifery, 2015. **31**(4): p. 400-408.

27. Huicho, L., et al., *How much does quality of child care vary between health workers with differing durations of training? An observational multicountry study.* Lancet, 2008. **372**(9642): p. 910-916.

28. Fulton, B.D., et al., *Health workforce skill mix and task shifting in low income countries: a review of recent evidence.* Hum Resour Health, 2011. **9**(1): p. 1.

29. Bogren, M.U., et al., *Towards a midwifery profession in Bangladesh–a systems approach for a complex world.* BMC Pregnancy Childbirth, 2015. **15**(1): p. 325.

30. UNFPA Bangladesh. *UNFPA's success in saving mothers and babies' lives in Bangladesh: 1,143 Midwives deployed to government health facilities*. 2018; Available from: https://bangladesh.unfpa.org/en/news/unfpas-success-saving-mothers-and-babies%E2%80%99-lives-bangladesh-1143-midwives-deployed-government.

31. Moxon, S.G., et al., *Service readiness for inpatient care of small and sick newborns: what do we need and what can we measure now?* J Glob Health, 2018. **8**(1).

32. Jayanna, K., et al., *Assessment of facility readiness and provider preparedness for dealing with postpartum haemorrhage and pre-eclampsia/eclampsia in public and private health facilities of northern Karnataka, India: a cross-sectional study.* BMC Pregnancy Childbirth, 2014. **14**(1): p. 304.

33. While, A.E., *Competence versus performance: which is more important?* J Adv Nurs, 1994. **20**(3): p. 525-531.

For peer review only - http://bmjopen.bmj.com/site/about/guidelines.xhtml

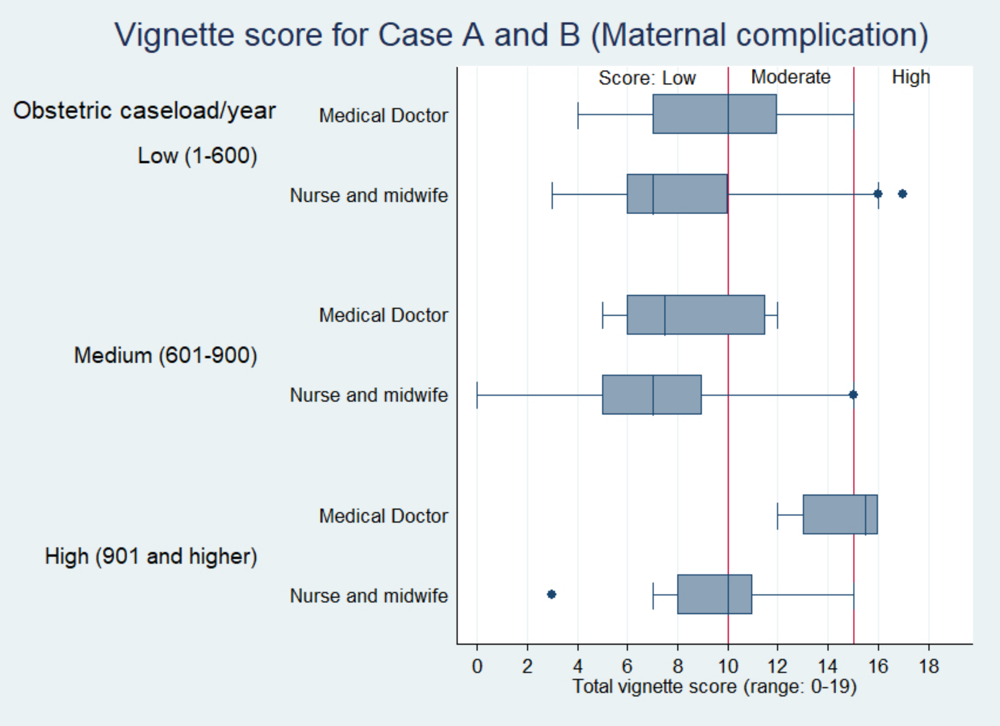

The vignette scores for obstetric complications by providers cadre and type of facility

72x52mm (300 x 300 DPI)

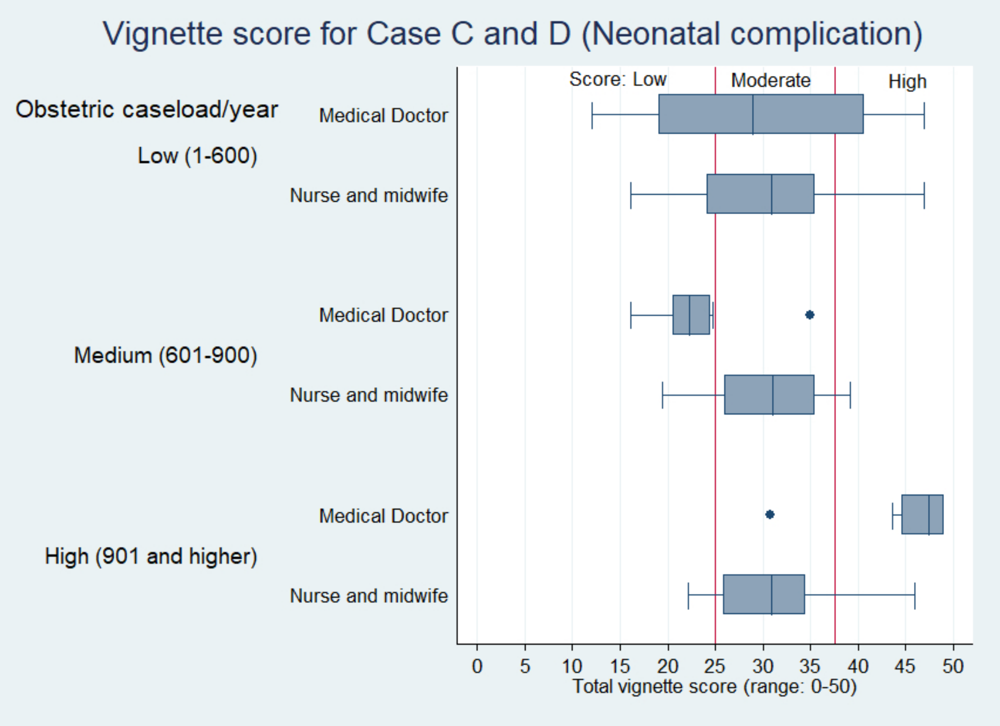

The vignette scores for neonatal complications by providers cadre and type of facility

72x52mm (300 x 300 DPI)

S1 Appendix: Obstetric and neonatal vignette scenarios

Case A (Eclampsia):

A 26-year-old woman who is 7 months pregnant comes in complaining of headaches, blurred vision and epigastric pain and her face looks swollen. In this facility, what would you usually do to establish a diagnosis?

1. Measure woman's blood pressure
2. Check urine for protein
3. Check her reflexes
4. Check fetal heart rate
5. Refer to other health facility immediately

Upon examination she had a blood pressure of 170/120, 3+ protein in her urine and brisk reflexes. How would she be managed at this facility?

1. Giving antihypertensive drug, e.g. hydralazine, labetalol, nifedipine
2. Give Magnesium sulfate or, if not available, diazepam
3. Give diuretics
4. Have somebody stay with her all the time in case she starts having seizures
5. Plan for delivery within the next 24 hours
6. Refer to other health facility immediately

Case B (Antepartum haemorrhage):

A 35-year old woman who is 8 months pregnant comes to this facility because she has started to bleed heavily vaginally. She has no contractions and does not complain of any pain. In this facility, what would you usually do to establish a diagnosis?

1. Check woman's vital signs
2. Check fetal heart rate
3. Perform abdominal examination
4. Will not perform vaginal examination

The woman has a feeble pulse at 120/min, her systolic blood pressure is 85 and she is pale, sweating and breathing rapidly at 30 breaths per minute. Foetal heart sound is normal. There is no tenderness on abdominal examination. She is still bleeding vaginally, bright red blood.

For peer review only

You suspect placenta praevia and therefore do not perform a vaginal examination. How would such a patient be managed now?

1. Elevate legs to increase return of blood to the heart
2. Give IV fluids rapidly
3. Give oxygen by mask or nasal cannula
4. Do ultrasound to confirm diagnosis
5. Prepare for Caesarian section
6. Give blood transfusion
7. Refer to hospital where Caesarean section can be done

Case C (Care for very low birth weight baby):

A 17-year-old woman pregnant for 8 months delivered a baby at home. The baby was weighed and found to be 1.4kg. The baby was referred to your facility and suppose you had admitted her for immediate care. What would you do for this baby?

1. Detain for thorough examination
2. Ensure breastfeeding is established and provide support if necessary
3. Put the baby in an incubator
4. Teach the mother to keep baby Skin-to-skin or KMC
5. Check cord dressing and other potential sources of infection
6. Encourage and ensure hygiene in care
7. Refer to a hospital/another facility

Mother says the baby is not breastfeeding and was contemplating giving glucose solution. What would you do?

1. Watch her breastfeed her baby and teach her good positioning and attachment
2. Examine the baby’s mouth to ensure there are no anatomical deformities
3. If baby not breast feeding, teach her to express the milk and feed with a clean cup
4. Encourage infant formula only if EBM is not possible and mother can afford
5. Educate her and encourage her to practice exclusive breastfeeding for 1st 6 months of the baby’s life

Case D (Essential newborn care and resuscitation):

A woman presented here in labour. The FHR is more than 160bpm. On examination, her cervix was fully dilated, and the baby had the head in the perineum. The baby is delivered and is normal weight but did not cry after delivery; what would you do for this baby?

For peer review only

1. Dry quickly
2. Examine and suction mouth
3. Ensure extra warmth for the baby
4. Use bag and mask to ventilate if baby does not cry after suctioning
5. Apply cardiac massage if ventilation alone does not help
6. Refer to another facility/hospital

Suppose the resuscitation was successful, what would you do next?

1. Initiate breastfeeding immediately
2. skin-to-skin contact with mother
3. Ensure and encourage hygiene

During routine checking on the baby after about 2 hrs, you saw the baby sleeping alone and the mother is sleeping but not in touch with baby. There was no covering on the baby since it wriggled out of the mother's cloth. What would you do?

1. Feel if baby is too cold
2. Take the temperature with a thermometer
3. Give SSC/KMC by mother or put in incubator for rewarming
4. Check the ward to see if windows are open and close them all as well as any fans on the ward to prevent draught
5. Let the mother breastfeed the baby immediately

For peer review only

Check-list: Revised Standards for Quality Improvement Reporting Excellence (SQUIRE 2.0) September 15, 2015

Text Section and Item Name	Section or Item Description	Page number
1. Title	Indicate that the manuscript concerns an initiative to improve healthcare (broadly defined to include the quality, safety, effectiveness, patient-centeredness, timeliness, cost, efficiency, and equity of healthcare)	1
2. Abstract	a. Provide adequate information to aid in searching and indexing	2
	b. Summarize all key information from various sections of the text using the abstract format of the intended publication or a structured summary such as: background, local problem, methods, interventions, results, conclusions	2
**Introduction**	**Why did you start?**	
3. Problem description	Nature and significance of the local problem	4
4. Available knowledge	Summary of what is currently known about the problem, including relevant previous studies	4
5. Rationale	Informal or formal frameworks, models, concepts, and/or theories used to explain the problem, any reasons or assumptions that were used to develop the intervention(s), and reasons why the intervention(s) was expected to work	4
6. Specific aims	Purpose of the project and of this report	4-5
**Methods**	**What did you do?**	
7. Context	Contextual elements considered important at the outset of introducing the intervention(s)	5

For peer review only

8. Intervention(s)	a. Description of the intervention(s) in sufficient detail that others could reproduce it	5
	b. Specifics of the team involved in the work	6
9. Study of the Intervention(s)	a. Approach chosen for assessing the impact of the intervention(s)	5-6
	b. Approach used to establish whether the observed outcomes were due to the intervention(s)	5-6
10. Measures	a. Measures chosen for studying processes and outcomes of the intervention(s), including rationale for choosing them, their operational definitions, and their validity and reliability	5-6
	b. Description of the approach to the ongoing assessment of contextual elements that contributed to the success, failure, efficiency, and cost	5-6
	c. Methods employed for assessing completeness and accuracy of data	6
11. Analysis	a. Qualitative and quantitative methods used to draw inferences from the data	6-7
	b. Methods for understanding variation within the data, including the effects of time as a variable	Not applicable
12. Ethical Considerations	Ethical aspects of implementing and studying the intervention(s) and how they were addressed, including, but not limited to, formal ethics review and potential conflict(s) of interest	7
**Results**	**What did you find?**	
13. Results	a. Initial steps of the intervention(s) and their evolution over time (e.g., time-line diagram, flow chart, or table), including modifications made to the intervention during the project	7-9

For peer review only

	b. Details of the process measures and outcome	10-12
	c. Contextual elements that interacted with the intervention(s)	7-9
	d. Observed associations between outcomes, interventions, and relevant contextual elements	11-12
	e. Unintended consequences such as unexpected benefits, problems, failures, or costs associated with the intervention(s).	Not applicable
	f. Details about missing data	Not applicable
**Discussion**	**What does it mean?**	
14. Summary	a. Key findings, including relevance to the rationale and specific aims	13
	b. Particular strengths of the project	13
15. Interpretation	a. Nature of the association between the intervention(s) and the outcomes	13-14
	b. Comparison of results with findings from other publications	13-14
	c. Impact of the project on people and systems	13-14
	d. Reasons for any differences between observed and anticipated outcomes, including the influence of context	13-14
	e. Costs and strategic trade-offs, including opportunity costs	Not applicable
16. Limitations	a. Limits to the generalizability of the work	15
	b. Factors that might have limited internal validity such as confounding, bias, or imprecision in the design, methods, measurement, or analysis	15
	c. Efforts made to minimize and adjust for limitations	15
17. Conclusions	a. Usefulness of the work	15-16

For peer review only

	b. Sustainability	16
	c. Potential for spread to other contexts	16
	d. Implications for practice and for further study in the field	16
	e. Suggested next steps	16
**Other information**		
18. Funding	Sources of funding that supported this work. Role, if any, of the funding organization in the design, implementation, interpretation, and reporting	16

For peer review only

Competence of the Health Care Professionals in Diagnosing and Managing Obstetric Complications and Conducting Neonatal Care: A Clinical Vignette Based Assessment in District and Sub-District Hospitals in Northern Bangladesh

Journal:	*BMJ Open*
Manuscript ID	bmjopen-2018-028670.R1
Article Type:	Research
Date Submitted by the Author:	24-Jun-2019
Complete List of Authors:	Khan, Abdullah Nurus Salam; University of South Carolina, Health promotion, education and behavior; International Centre for Diarrhoeal Disease Research Bangladesh, Maternal and Child Health Division<br>Karim, Farhana; International Centre for Diarrhoeal Disease Research Bangladesh, Maternal and Child Health Division<br>Chowdhury, Mohiuddin Ahsanul Kabir; University of South Carolina, Epidemiology; International Centre for Diarrhoeal Disease Research Bangladesh, Maternal and Child Health Division<br>Zaka, Nabila; UNICEF USA,<br>Manu, Alexander; Liverpool School of Tropical Medicine<br>Arifeen, Shams E.; International Centre for Diarrhoeal Disease Research Bangladesh, Maternal and Child Health Division<br>Billah, Sk; International Centre for Diarrhoeal Disease Research, Maternal and Child Health Division
**Primary Subject Heading**:	Health services research
Secondary Subject Heading:	Evidence based practice, Health policy, Public health
Keywords:	Human resource management < HEALTH SERVICES ADMINISTRATION & MANAGEMENT, Quality in health care < HEALTH SERVICES ADMINISTRATION & MANAGEMENT, Organisation of health services < HEALTH SERVICES ADMINISTRATION & MANAGEMENT, OBSTETRICS, PERINATOLOGY, PUBLIC HEALTH

SCHOLARONE™
Manuscripts

Title

Competence of the Health Care Professionals in Diagnosing and Managing Obstetric Complications and Conducting Neonatal Care: A Clinical Vignette Based Assessment in District and Sub-District Hospitals in Northern Bangladesh

Authors

Abdullah Nurus Salam Khan[1,2], Farhana Karim[1], Mohiuddin Ahsanul Kabir Chowdhury[1,2], Nabila Zaka[3], Alexander Manu[4], Shams El Arifeen[1], Sk Masum Billah[1]

[1] Maternal and Child Health Division, International Centre for Diarrhoeal Disease Research, Bangladesh (icddr,b), Dhaka, Bangladesh

[2] Arnold School of Public Health, University of South Carolina

[3] Health Section, Maternal and Newborn Health, UNICEF, New York, USA

[4] Centre for Maternal and Newborn Health, Liverpool School of Tropical Medicine, Liverpool, UK

# Corresponding author

Name: Sk Masum Billah

Address: Maternal and Child Health Division, International Centre for Diarrhoeal Disease Research, Bangladesh (icddr,b);
68 Shahid Tajuddin Ahmed Sarani, Mohakhali, Dhaka 1212, Bangladesh

Telephone: +880 171 7416117

Fax: +880 298 27077

Email: billah@icddrb.org

For peer review only - http://bmjopen.bmj.com/site/about/guidelines.xhtml

Abstract

Background: This study assesses the competency of maternal and neonatal health (MNH) professionals at district and sub-district level health facilities in northern Bangladesh in managing maternal and newborn complications using clinical vignettes. The study also examines whether the professional's characteristics and provision of MNH services in health facilities influence their competencies.

Methods: 134 MNH professionals in 15 government hospitals were interviewed during August and September 2016 using structured questionnaire with clinical vignettes on obstetric complications (antepartum haemorrhage and pre-eclampsia) and neonatal care (low-birthweight and immediate newborn care). Summative scores were calculated for each vignette and median scores were compared across different individual and facility level attributes to examine their association with competency score. Kruskal-Wallis test was performed to identify the significance of association considering p-value<0.05 as statistically significant.

Results: The competency of MNH professionals was low; About 10% and 24% of the health professionals received 'high' score (>75% of total) in maternal and neonatal vignettes, respectively. Medical doctors had higher competency than nurses and midwives (score=11 vs. 8 out of 19, respectively; p=0.0002) for maternal vignettes, but similar competency for neonatal vignettes (score=30.3 vs. 30.9 out of 50, respectively). Professionals working in health facilities with higher utilization of normal deliveries had better competency than their counterparts. Professionals had higher competency in newborn vignettes (significant) and maternal vignettes (statistically not significant) if they worked in health facilities that provided more specialized newborn care services and emergency obstetric care, respectively, in last 6 months.

Conclusions: Despite the overall low competency of MNH professionals, exposure to a higher number of obstetric cases at the workplace was associated with their competency. Arrangement of periodic skill-and-drill based in-service training for MNH professionals in high-utilization neighbouring health facilities could be a feasible intervention to improve their knowledge and skill in obstetric and neonatal care.

For peer review only - http://bmjopen.bmj.com/site/about/guidelines.xhtml

Strengths and limitations

- The study assesses the competence (knowledge and skill) of doctors, nurses, and midwives in managing complicated obstetric and neonatal cases using clinical vignettes
- The study identifies the association of health care professional's competency with individual-level factors such as cadre type, work experience, training status, and health facility level factors such as health facility type, provision of maternal and neonatal health services, and obstetric caseload
- The study highlights the need for local and national health authorities to prioritize continuous professional development and practical clinical training to assure a fully competent and skilled birth attendance in low-resource settings
- The study does not validate the competency assessed by clinical vignettes by comparing it with direct clinical observation or chart abstraction of performed services at clinical settings

For peer review only

For peer review only - http://bmjopen.bmj.com/site/about/guidelines.xhtml

Background

Maternal and neonatal survival has received significant priorities in the global agenda of Millennium Development Goals (MDG), and subsequently in Sustainable Development Goals (SDG) [1-3]. During the MDG era, the maternal mortality ratio (MMR) and the neonatal mortality rate (NMR) have been reduced by 44% and 49%, respectively, between 1990 and 2015 [4, 5]. Despite the progress achieved in reducing MMR and NMR, 303,000 mothers and 4 million newborns are still estimated to be dying each year worldwide and 99% of them are dying in developing regions of the world [4, 5]. A WHO systematic review finds that around 27% and 14% of all maternal death is due to haemorrhage and complications arising from hypertensive disorders of pregnancy including preeclampsia, respectively [6]. For newborns, the major reasons for mortality are preterm birth, birth asphyxia and infections [7, 8]. The evidence indicates that the highest mortality risk for both mothers and their babies is on the first day of birth [9]. It is estimated that 16 to 33% of maternal mortality can be averted if the major causes of death can be managed timely and efficiently by skilled attendance during childbirth [10]. The mere presence of skilled birth attendant in health facilities equipped with key signal functions of emergency obstetric care, however, does not ensure the best practices of intrapartum and immediate postpartum care. To improve the survival of mothers and newborns through the best practices, health professional's competence to diagnose and manage complications arising at the time of childbirth is strongly required as the low and middle-income countries (LMIC) are struggling to increase the skilled birth attendance for every childbirth [11, 12]. The knowledge and skill gap of the skilled birth attendants in LMICs often remain unaddressed as the countries have limited resources for assessing and improving the competencies of MNH professionals [13, 14].

Clinical vignette or written case simulation is an inexpensive and validated way to reflect on performance and quality of health care professionals' actual practice [15, 16] especially in low-income settings where chart documentation is often incomplete for obstetric cases [17, 18]. Assessment based on clinical vignettes is more feasible and accurate than direct clinical observation of health professional's performance where actual performance could be limited by the shortage of essential drugs, equipment or supplies and infrequent presentation of critical obstetric cases (<15% of all pregnancies) [19, 20]. This paper aims to assess the competency of health care professionals at district and sub-district level health facilities in northern Bangladesh in managing maternal and newborn complications using clinical

For peer review only - http://bmjopen.bmj.com/site/about/guidelines.xhtml

vignettes. The clinical vignettes are adopted from two studies conducted in Ghana as part of the overall quality of care evaluation of hospital-based MNH services [21, 22]. The authors found overall moderate competency of MNH workers and its association with demand for facility deliveries, availability of infrastructure, and workload, however, did not explore the association with different attributes of health care professionals in detail. Independent of focusing on another LMIC context, this paper also explores whether individual-level factors such as cadre type, training on emergency obstetric and neonatal care, work experience and health facility-level factors such as health facility type, obstetric caseloads i.e. annual delivery conducted in health facilities, and provision of emergency obstetric and neonatal care are associated with the competence of the health professionals. Findings from this paper will be useful in designing capacity development programmes for MNH professionals and bringing procedural changes in health facilities of LMICs to improve maternal and neonatal health outcomes through evidence-based skilled childbirth services.

Methodology

Study design and study sites:

This study draws on the findings from a cross-sectional assessment conducted for the baseline evaluation of "Every Mother Every Newborn Quality Improvement (EMEN QI)" initiatives, jointly implemented by Government of Bangladesh, UNICEF headquarter and UNICEF Bangladesh. The initiative focuses on implementing quality of care standards for maternal and neonatal health care delivery in the selected health facilities of three neighbouring districts– Kurigram, Lalmonirhat, and Gaibandha, situated in the northern part of Bangladesh. From each of the districts, four sub-district health facilities or Upazila Health Complex (UHC) and one district hospital (DH) were selected. All UHCs are primary level referral facilities designated to provide at least basic emergency obstetric and newborn care (BEmONC). The DHs, secondary level health facilities, offer comprehensive emergency obstetric and newborn care (CEmONC) services. The data were collected between August and September 2016.

Selection of MNH care professionals:

From 3 DHs and 12 UHCs, a total of 134 health care professionals who were posted and present at these health facilities on the assessment day and were responsible to conduct

For peer review only - http://bmjopen.bmj.com/site/about/guidelines.xhtml

normal deliveries, manage obstetric or neonatal emergencies were selected for the assessment. The 134 respondents included 39 medical doctors, 91 nurses and 4 midwives.

Administration of vignettes:

We used two vignettes (case A and B) to describe obstetric complications and two vignettes (case C and D) on newborn care. Case A and B describe scenarios on pregnant women with signs of preeclampsia and antepartum haemorrhage, respectively, and both cases include separate sections on 'diagnosis' and 'management' of those conditions. Case C describes a baby with foetal distress and comprises three parts- i) resuscitation ii) immediate newborn care, and iii) thermal care. Case D is about very low birth weight baby and comprises two parts – i) immediate management, and ii) counselling caregivers on breastfeeding.

A team of trained research physicians administered all four clinical vignettes among the health professionals through face-to-face interview using a structured questionnaire including a check-list. At first, the research physician read out the scenarios to the health care professionals and asked for specific actions to be taken for each of the scenarios. It was done one scenario at a time up to where the action was required. Responses made by the health professionals to each of the scenarios were then matched with the pre-defined vignette action points in the check-list (Appendix S1). The vignette action points were based on the WHO's Integrated Management of Pregnancy and Childbirth (IMPAC) guidelines for the selected obstetric and neonatal cases [23]. Responses were considered as correct if they were in agreement to that in the list. The research physicians were trained on the vignette scenarios and the definition of each vignette action points. Since all four vignette scenarios were read out and responses were collected for each, there was no incomplete data. Intra- and inter-observer variability in recording the responses were less likely to occur as the responses were structured.

Assessing the provision of MNH services:

In 15 health facilities, a separate team of research assistants extracted the information on the number of conducted deliveries and the provision of emergency obstetric care and specialized newborn services in the last 6 months from health facility records using a checklist. Emergency obstetric care included administration of injectable antibiotics, injectable oxytocin, injectable anticonvulsant; manual removal of retained placenta, retained products of conception, and provision of assisted vaginal delivery, blood transfusion, and caesarean delivery. Specialized newborn services were the provision of kangaroo mother care (KMC),

For peer review only - http://bmjopen.bmj.com/site/about/guidelines.xhtml

basic resuscitation (clearing of the airway, stimulation, bag and mask ventilation), advanced resuscitation (intubation, chest compression), administration of oxygen, intravenous fluid, and antenatal corticosteroid.

Data analysis:

To analyse the total vignette score obtained by the respondents, each of the 'actions' was assigned to either equal score (1 point for each action point listed for case A and B) or weighted scores (for case C and D). The detailed methodology of assigning the scores are mentioned elsewhere [21, 22] and the exact scores for each action points used in analysis are listed in Appendix S1. The authors mentioned in their papers that they had asked expert opinions on the relative importance of the actions for all four vignette cases to assign weighted scores for each action point. Based on the post-hoc analysis the authors decided to keep the simpler approach of assigning equal score for maternal vignettes (case A and B) as there were no major differences in results between equal or weighted scores [21], however, chose weighted scoring for neonatal vignettes (case C and D) [22].

Based on the assigned scores, summative scores were calculated for the vignette cases on maternal and neonatal complications. Vignette scores were compared between different categories of individual and health facility level attributes using Kruskal-Wallis tests. The scores were further categorized into 'high' if the health professional received more than 75% of the total score, 'moderate' for 50-75% of the total, and 'low' for less than 50% of the total. Health care professionals were categorized based on their cadre types, total working experience, training status, and whether they were placed in maternal or neonatal unit in the health facility; and health facilities were categorized based on their type (district or sub-district), annual case-load of deliveries and provision of emergency management for maternal and neonatal complications. The average number of annual deliveries conducted in the health facilities is presented in mean with standard deviation. Based on the average case-load of deliveries, the health facilities were further categorized into 'low' utilization if they conducted less than 600 deliveries a month on an average, 'medium' (600-900 deliveries), and 'high' (more than 900 deliveries a month). The cut-off points were set roughly matching with the expected number of institutional births in EmONC equipped health facilities based on population size and expected number of pregnancies in Bangladesh [24], however, the range does not represent the national or regional estimate of institutional deliveries in district or sub-district hospitals. Health facility-level service provision indicators were considered

For peer review only - http://bmjopen.bmj.com/site/about/guidelines.xhtml

'available' only if the activities related to these indicators were performed within six months prior to the assessment and reported using frequency and percentage of the health facilities providing them. A p-value less than 0.05 was considered as statistically significant for the Kruskal-Wallis test. The analysis was performed using Stata 13.0.

Patient and public involvement:

The research questions and outcome measures were related to assessing the knowledge and skill of government health care professionals in managing obstetric and neonatal complications using clinical vignettes. In the selection of cases for clinical vignettes, the critical cases were chosen and presented as they would occur among pregnant women and their newborn in the context of Bangladesh. Patients or pregnant women were not involved in the design of, in the recruitment to and conduct of the study. The baseline results were disseminated among the health managers and MNH professionals in the government hospitals where the study was conducted and the endline results will be disseminated in due course.

Ethical approval:

The Ethical Review Committee (ERC) of icddr,b approved the study (protocol number PR-16024). Informed written consent was sought from the health care professionals and health facility managers before conducting the interviews.

Results

Facility delivery utilization and provision of emergency obstetric and specialized neonatal care:

Table 1 presents the status of delivery utilization and service provision of the surveyed health facilities regarding emergency obstetric and newborn care by health facility types- district and sub-district. During preceding three years since the assessment, average number of deliveries conducted in a year was less than 600 (low-utilization) in 10 out of 12 sub-district hospitals, whereas in two out of three district hospitals and one sub-district hospital the average number was more than 900 a year (high-utilization) and rest two of the 15 health facilities were between 600-900 a year (medium-utilization). On average, the district hospitals had higher obstetric caseloads than the sub-district hospitals (908 ±301 vs. 410±268 annual deliveries). All signal functions of comprehensive emergency obstetric care (CEmOC) but 'assisted vaginal delivery' were performed in all three district hospitals within 6 months

For peer review only - http://bmjopen.bmj.com/site/about/guidelines.xhtml

222 prior to the assessment. Injectable anticonvulsant and blood transfusion were provided and caesarean deliveries were performed in 42%, 25% and 8% of the 12 sub-district hospitals, respectively. Kangaroo mother care (KMC) was not practised in any of the health facilities. For birth asphyxia management, basic resuscitation procedures such as airway clearing, stimulation and bag-mask ventilation were practised in all the health facilities within 6 months prior to the assessment, but advanced resuscitation measures such as intubation or chest compression were only conducted in two district- and two sub-district hospitals within the same period.

230 Table 1: Annual delivery utilization, and provision of emergency obstetric and specialized neonatal care in the health facilities within 6 months prior to the assessment

**Indicators**	**Total, n(%)**	**District hospital, n(%)**	**Sub-district hospital, n(%)**
Total number	15	3	12
**Annual delivery conducted on average**			
Mean (±SD)	509 (±335)	908 (±301)	410 (±268)
Low; <600	10 (66.7)	0 (0.0)	10 (83.3)
Medium; 600-900	2 (13.3)	1 (33.3)	1 (8.3)
High; >900	3 (20.0)	2 (66.7)	1 (8.3)
**Provision of emergency obstetric care**			
Injectable antibiotics administration	15 (100.0)	3 (100.0)	12 (100.0)
Injectable oxytocin administration	15 (100.0)	3 (100.0)	12 (100.0)
Injectable anticonvulsant administration	8 (53.3)	3 (100.0)	5 (41.7)
Manual removal of retained placenta	14 (93.3)	3 (100.0)	11 (91.7)
Manual removal of retained products of conception	14 (93.3)	3 (100.0)	11 (91.7)
Assisted vaginal delivery	0 (0.0)	0 (0.0)	0 (0.0)
Blood transfusion services	6 (40.0)	3 (100.0)	3 (25.0)
Caesarean delivery	4 (26.7)	3 (100.0)	1 (8.3)
**Provision of specialized care for newborn**			
Provision of kangaroo mother care (KMC)	0 (0.0)	0 (0.0)	0 (0.0)
Basic resuscitation[1]	15 (100.0)	3 (100.0)	12 (100.0)
Advanced resuscitation[2]	4 (26.7)	2 (66.7)	2 (16.7)
Oxygen administration	15 (100.0)	3 (100.0)	12 (100.0)

For peer review only - http://bmjopen.bmj.com/site/about/guidelines.xhtml

IV fluid administration	14 (93.3)	3 (100.0)	11 (91.7)
Antenatal corticosteroid (ACS) administration	3 (20.0)	2 (66.7)	1 (8.3)

[1] Clearing of airway, stimulation, bag and mask ventilation

[2] Intubation, chest compression

Characteristics of health care professionals:

The mean (±SD) age of 134 health professionals was 37.7 (±7.5) years, however, the mean age of nurses and midwives was higher than the medical doctors (40.0±6.9 vs. 32.2±6.1 years). About one-third of doctors and all the nurses and midwives were female (table 2). Among the interviewed health professionals, 84% and 62% were working in maternity and neonatal wards, respectively. For half of the health professionals both working areas overlapped as they had experience of working in both wards. None of the medical doctors had more than five years of working experience whereas 59% of the nurses and midwives had so. A higher proportion of nurses and midwives had ever received an in-service training on obstetric complications compared to the doctors (96% vs. 44%) and about 45% of all health professionals had ever received an in-service training on newborn complications. Within 12 months prior to the assessment, only 7% and 29% of health professionals received any training on obstetric and newborn complications, respectively.

Table 2: Characteristics of health professionals

**Characteristics**	**Total, %**	**Medical doctor, %**	**Nurse and midwife, %**
Total number	134	39	95
**Age in years**			
Less than 30	21.6	59.0	6.3
31- 40	48.5	33.3	54.7
41- 50	21.6	2.6	29.5
More than 50	8.2	5.1	9.5
Female health professionals	79.9	30.8	100.0
**Type of health facility where posted**			
District hospital	24.6	17.9	27.4
Sub-district hospital	75.4	82.1	72.6
**Majorly works in**			
Both maternity and neonatal wards	51.5	56.4	49.5
Maternity ward	83.6	87.2	82.1
Neonatal ward	61.9	61.5	62.1
**Work experience in years**			
Less than 1	8.2	17.9	4.2

For peer review only

1-2	38.8	71.8	25.3
3-5	11.2	10.3	11.6
More than 5	41.8	0.0	58.9
**Ever received in-service training on**			
Management of obstetric complication	80.6	43.6	95.8
Management of newborn complication	44.8	51.3	42.1
**Received training in the last 12 months on**			
Management of obstetric complication	6.7	5.1	7.4
Management of newborn complication	29.1	28.2	29.5

Competence of health professionals in diagnosing and managing maternal and neonatal complications:

For vignettes dealing with obstetric complications (case A and B), the median summative score was 8 with interquartile range (IQR) of 6 to 11 (table 3). About 60% of the health professionals received ‘low’ score i.e. less than 50% of the maximum possible score and only 10% achieved ‘high’ score (more than 75% of the total score). Between the two obstetric cases, health professionals had slightly better knowledge about diagnosis and management of eclampsia (case A) than antepartum haemorrhage (APH) (case B) (49% received ‘moderate’ to ‘high’ score vs. 38%, respectively). Moreover, 51% of the health professionals had ‘moderate’ to ‘high’ score regarding the diagnosis of APH and eclampsia in comparison to 36% who had the similar competency regarding the management of these cases.

The median summative vignette score for neonatal complications (case C and D) was 30.8 (IQR: 23.9, 36.6) where 50 was the maximum possible score. About 70% of the health professionals could achieve at least 50% of the total score in newborn vignettes. Between the two cases, health professionals’ competence on provision of essential newborn care (case D) was better than that for management of very low birth weight baby (case C) as evident from the categorical classifications of scores (78% received ‘moderate’ to ‘high’ score vs. 58%, respectively).

Table 3: Median vignette scores and their categorical classifications

Vignette cases	Total range	Median score (IQR)	Categorical score (%), N=134		
			High (>75%)	Moderate (50-75%)	Low (<50%)
**Maternal complication**					
Case A (Eclampsia)	0-09	4 (3,6)	12.7	36.6	50.7
Case B (Antepartum haemorrhage)	0-10	4 (2,6)	8.2	29.9	61.9
Case A+B (Diagnosis component)	0-09	5 (3,6)	11.2	40.3	48.5
Case A+B (Management component)	0-10	4 (2,6)	8.2	27.6	64.2

For peer review only - http://bmjopen.bmj.com/site/about/guidelines.xhtml

Case A+B (Total)	0-19	8 (6,11)	10.4	29.9	59.7
**Neonatal complication**					
Case C (Care for VLBW babies)	0-20	11.1 (8.4, 14.9)	23.9	34.3	41.8
Case D (Essential newborn care)	0-30	19.8 (15.6,24.4)	30.6	47.0	22.4
Case C+D (Total)	0-50	30.8 (23.9, 36.6)	23.9	46.3	29.9

IQR= Interquartile range; VLBW= Very low birth weight

Table 4 presents the distribution of median vignette scores for maternal and neonatal complications across different attributes of the health professionals and their working health facilities. For maternal complications, the median scores differed significantly between the two cadres of health professionals (p=0.0002) with medical doctors achieving a higher score than nurses or midwives. Neither of the maternal or neonatal vignette scores was significantly different among the health professionals who worked in both maternal and neonatal wards or only in one of these wards or between two levels of health facilities. Health professionals' duration of work experience or their receipt of training on management of obstetric or neonatal complication did not make any significant difference in their competencies. Health professionals working in 'high' utilization health facilities with more than 900 annual deliveries had significantly better competency in diagnosing and managing both maternal and neonatal complications than their counterparts (p=0.0005 and p=0.004, respectively). Health professionals working in health facilities which provided more than 5 out of 7 EmOC services had statistically non-significant (p=0.6819) but higher competency in maternal vignettes. Similarly, health professionals had significantly higher competency in newborn vignettes (p=0.0361) if they were working in health facilities which were better equipped to provide emergency management for newborn complications.

Table 4: Median scores of maternal and neonatal vignettes across the categories of individual and health facility level attributes

**Characteristics**	**Maternal complication (Case A and B)**		**Neonatal complication (Case C and D)**	
	**Median score[1] (IQR)**	**p-value[2]**	**Median score[1] (IQR)**	**p-value[2]**
**Type of health professionals**				
Medical doctor	11 (7, 14)	**0.0002**	30.3 (21.0, 45.3)	0.6682
Nurse and midwives	8 (6, 10)		30.9 (25.5, 35.5)	
**Majorly works in**				
Both maternity and neonatal ward	9 (6, 11)	0.7540	30.9 (24.1, 36.0)	0.3636
Maternity ward only	8 (6, 11)		27.9 (23.0, 36.6)	
Neonatal ward only	8 (7, 13)		32.3 (26.0, 39.2)	
**Work experience**				
Less than 1 year	10 (7, 12)	0.1099	30.9 (23.3, 37.5)	0.8443

For peer review only - http://bmjopen.bmj.com/site/about/guidelines.xhtml

1-5 years	9 (7, 12)		31.6 (22.1, 39.2)	
More than 5 years	7.5 (5, 10)		29.7 (25.7, 35.3)	
**Ever trained on management of obstetric complication**				
Yes	8 (6, 11)	0.0931	30.9 (24.5, 36.1)	0.6628
No	9 (7, 13)		29.5 (20.5, 45.3)	
**Ever trained on management of neonatal complication**				
Yes	8.5 (5, 11)	0.8005	30.8 (21.8, 36.3)	0.3817
No	8 (6, 12)		30.8 (25.9, 37.6)	
**Received training in last 12 months on the management of obstetric complication**				
Yes	10 (6, 12)	0.5456	30.3 (27.6, 36.3)	0.3789
No	8 (6, 11)		30.7 (23.3, 36.6)	
**Received training in last 12 months on the management of neonatal complication**				
Yes	9 (6, 12)	0.3674	31.9 (23.0, 37.5)	0.5567
No	8 (6, 11)		30.1 (24.3, 36.6)	
**Type of health facility where posted**				
District hospital	8 (7, 11)	0.6682	28.7 (25.5, 35.5)	0.4618
Sub-district hospital	8 (6, 11)		31.3 (23.3, 37.7)	
**Annual delivery conducted in the health facility**				
Low; <600	8 (6, 11)	**0.0005**	30.0 (22.9, 36.3)	**0.004**
Medium; 600-900	7 (5, 9)		27.4 (23.3, 34.3)	
High; >900	12 (9, 15)		34.5 (30.2, 46.1)	
**Provision of EmOC[3]**				
5 or less services	8 (6, 12)	0.6819	31.3 (23.2, 38.2)	0.4164
More than 5	9 (7, 11)		29.1 (25.5, 35.5)	
**Provision of emergency management for neonatal complication[4]**				
3 or less services	8 (6, 11)	0.2722	29.3 (23.0, 35.7)	**0.0361**
More than 3	8 (7, 12.5)		31.8 (26.0, 38.7)	

[1] Score range for maternal complication is 0-19 and for neonatal complication, the range is 0-50

[2] p-value is obtained from the Kruskal-Wallis test

[3] Provision of injectable antibiotic, oxytocin, and anticonvulsant; Manual removal of placenta and retained products, performing blood transfusion and caesarean-sections within 6 months prior to the assessment

[4] Provision of kangaroo mother care (KMC), basic and advanced resuscitation, administration of oxygen, intravenous fluids, and antenatal corticosteroids within 6 months prior to the assessment

Competency of health care professionals by obstetric case-loads of the health facilities:

For peer review only

For peer review only - http://bmjopen.bmj.com/site/about/guidelines.xhtml

Figure 1 and Figure 2 show the vignette scores for obstetric and neonatal complications, respectively, across the two cadres of health professionals by health facility's annual delivery utilization. The competence of the health professionals working in low utilization health facilities fall majorly in 'low' to 'moderate' categories and the median scores do not differ substantially between medical doctors and nurses or midwives. Medical doctors working in 'high' utilization health facilities show 'high' competency compared to most nurses or midwives, especially in newborn complication management.

Figure 1: The vignette scores for obstetric complications by health professional cadre and type of health facility

Figure 2: The vignette scores for neonatal complications by health professional cadre and type of health facility

Discussion

The study finds low competency of diagnosing and managing critical obstetric and neonatal conditions among the health professionals at district and sub-district hospitals in northern Bangladesh. Inadequate provision of health services for management of obstetric emergencies or specialized newborn care in the health facilities along with low coverage of recent refresher training might have reflected in low competencies of the MNH professionals at these hospitals. The competency, as measured by the vignette scores, varied significantly between the cadres of health professionals and was associated with obstetric caseloads of the health facilities.

All health care professionals were working in obstetric or neonatal wards in the selected health facilities and were expected to achieve standard competencies for the first line management of the scenarios presented in both the maternal and the newborn vignettes [24]. The vignette scores indicate that majority of the professionals achieved less than 50% of the total score i.e. 'low' competency for the obstetric complications- antepartum haemorrhage (APH) and eclampsia but showed better competencies for newborn complications. This could result from the poor retention of knowledge of managing obstetric complication due to lack of recent training on this topic. The data show that the proportion of health professionals receiving a training within the last 12 months on the management of maternal complication was lower than those receiving training on neonatal complications (7% vs. 30%) whereas most of them had ever received in-service training on these topics. Although capacity

For peer review only - http://bmjopen.bmj.com/site/about/guidelines.xhtml

For peer review only

development through targeted training of the maternal, newborn and child health (MNCH) care professionals is identified as a critical intervention to improve health outcome [25], this often fails to meet the desired outcome due to inadequate post-training monitoring, follow-up and periodic refresher training [26]. One study in India tested the feasibility and effectiveness of skill-and-drill based training for MNCH workers at sub-district and district level health facilities and found improved knowledge and skill of the health workers following the intervention [27]. The authors, however, emphasized on bringing system-level improvement i.e. ensuring appropriate infrastructure and equipment to translate the improved competency into actual practices. The study also identified that both vignette scores were higher among the health professionals who worked in health facilities with 'high' caseload of conducting deliveries but remained nearly similar in 'low' and 'medium' utilization health facilities. This association further underscores the fact that experiential learning or on-the-job experiences can contribute to the competence achieved through academic training only [28, 29]. The comprehensive package of training for emergency obstetric and newborn care (EmONC) could, therefore, include skill-and-drill based in-service training. The same health facility where the health professionals are posted or a neighbouring high utilization health facility can serve as an 'obstetric-skill laboratory' or 'simulation centre' for conveying this type of training periodically [30]. Operations researches should further explore effective and efficient ways to incorporate such interventions into the government's existing EmONC training programmes in low-resource settings of the LMICs.

Our study also identifies that the doctors had significantly better knowledge score than nurses and midwives in maternal complication vignettes but similar score in newborn vignettes. With the higher extent of academic and clinical training, it is not surprising that doctors would have higher competency score than nurses and midwives in the maternal vignettes which, in general, requires more intensive clinical procedures than the newborn scenarios. Their similar competence in newborn vignettes, however, indicates that advanced pre-service clinical training of the health professionals does not necessarily translate into better knowledge and skill, and similar evidence was found for sick child management in a multi-country study [31]. This directs the policymakers to consider the role of task-shifting from doctors to nurses and midwives to maximize the productive efficiency in health facilities of LMICs particularly for low intensive procedures like immediate newborn care or identification of obstetric complications and referral [32, 33]. This calls for the timely

For peer review only - http://bmjopen.bmj.com/site/about/guidelines.xhtml

initiative as in Bangladesh the newly deployed midwifery cadre is expected to work with their full capacity from 2016 and onwards according to the Midwifery Act [34].

The study found that median maternal vignette scores were higher, although statistically not significant, among the health professionals who worked in health facilities that were better equipped to provide emergency obstetric services whereas for newborn vignettes the scores were significantly higher in health facilities that could provide better newborn specialized services. This indicates that inadequate service provision for emergency management in the health facilities could result in low competency of the MNH professionals through infrequent exposure to critical cases. In the assessed sub-district hospitals, blood transfusion which is required in case of severe APH or injectable magnesium sulphate for early stabilization of pre-eclampsia was infrequently provided in the last six months. The gap was also profound for conducting caesarean sections, providing advanced resuscitation for newborn, and administering antenatal corticosteroid for premature labour. Kangaroo mother care (KMC), evidence-based care for low birth weight babies, was not provided in any of the assessed health facilities. The literature suggests that the competence of health professionals is shaped from actively engaging in evidence-based practices [29] and to implement such practices, the readiness of the health facilities should be optimum for carrying out the intended procedures [35, 36]. Improving the competence of health care professionals to ensure the procedural quality of care should, therefore, require structural improvement of the health facilities to allow them to conduct the required tasks.

The findings of the study should be cautiously interpreted as the study assesses the competence using clinical vignettes which explores what one should do at an expected level, not what one actually performs in real settings [37]. Clinical vignettes should be considered as a cheap alternative to direct clinical observation for assessing knowledge and skill of health professionals or where chart abstraction is not feasible due to poor chart documentation of obstetric cases [16]. The study objectives do not also consider whether the competency gap of MNH professionals translate into poor quality of maternal and neonatal care in the selected health facilities. Further research should focus on validating the vignette-based findings by examining the association of health professionals' competency with their actual practices and the related health outcomes for mother and newborn in the health facilities. The first level health facilities are not included in the study as they are not equipped or entitled to provide EmONC services and monthly utilization of normal delivery is very low

For peer review only - http://bmjopen.bmj.com/site/about/guidelines.xhtml

[24]. The study findings on health professionals' competence are therefore limited to district and sub-district level health facilities only.

Conclusion

Clinical vignettes could be a useful tool to assess the competency, a key predictor of performance, of MNCH professionals in low resource settings. Health professionals' cadre and obstetric case-load of the working health facilities were associated with their competence in diagnosing and managing maternal and newborn complications. The arrangement of periodic skill-and-drill based in-service and refreshers training using high-utilization neighbouring health facilities as 'obstetric skills laboratory' could be a feasible intervention to improve the competence of the MNCH professionals in LMICs.

For peer review only

For peer review only - http://bmjopen.bmj.com/site/about/guidelines.xhtml

Author's contribution

SEA, SMB, NZ, and MAKC designed the study and SMB, MAKC, and ANSK conceptualized the paper. ANSK, FK, and MAKC were involved in data acquisition and literature review. ANSK and FK conducted the analysis of the paper and NZ, AM, and SEA guided additional analysis. ANSK and FK prepared the first draft. MAKC, NZ, AM, SEA, and SMB contributed to revision and preparation of the final draft. All authors have reviewed and approved the final manuscript.

Competing interests

No conflict of interest is declared by any author.

Funding

The study was conducted with funding support from Bill and Melinda Gates Foundation (BMGF) for EMEN Quality Improvement initiative to UNICEF Headquarter (BMGF grant number OPP1112117, UNICEF and BMGF partnership grant).

Availability of data and material

The data that support the findings of this study are available from the corresponding author upon reasonable request.

Acknowledgement

We acknowledge the support received from Quality Improvement Secretariat (QIS) of Ministry of Health and Family Welfare and UNICEF Bangladesh to facilitate the data collection. We are grateful to the research participants i.e. the health care professionals, and hospital managers for allowing us to conduct the study. We are also thankful to the data collection and entry team for their commitment and sincere effort.

For peer review only - http://bmjopen.bmj.com/site/about/guidelines.xhtml

References

1. Sachs, J.D., *From millennium development goals to sustainable development goals.* Lancet, 2012. **379**(9832): p. 2206-2211.
2. WHO, U. and C. Mathers, *Global strategy for women's, children's and adolescents' health (2016-2030).* Organization, 2017. **2016**(9).
3. United Nations. *Sustainable development goals: 17 goals to transform our world.* 2015; Available from: https://www.un.org/sustainabledevelopment/sustainable-development-goals/.
4. World Health Organization, *Trends in maternal mortality: 1990 to 2015 Estimates by WHO, UNICEF, UNFPA, World Bank Group and the United Nations Population Division*. 2015: Geneva, Switzerland.
5. United Nations Inter-agency Group for Child Mortality Estimation (UN IGME). *Levels & trends in child mortality: Report 2017, Estimates developed by the UN Inter-agency Group for Child Mortality Estimation*. 2017: United Nations Children's Fund, New York.
6. Say, L., et al., *Global causes of maternal death: a WHO systematic analysis.* Lancet Glob Health, 2014. **2**(6): p. e323-e333.
7. Liu, L., Johnson, H. L., Cousens, S., Perin, J., Scott, S., Lawn, J. E., & Black, R. E. , 379, *Global, regional, and national causes of child mortality: an updated systematic analysis for 2010 with time trends since 2000.* Lancet, 2012. **379**(9832): p. 2151-2161.
8. Lawn, J.E., et al., *4 million neonatal deaths: when? Where? Why?* Lancet, 2005. **365**(9462): p. 891-900.
9. Ronsmans, C., W.J. Graham, and Lancet Maternal Survival Series steering group., *Maternal mortality: who, when, where, and why.* Lancet, 2006. **368**(9542): p. 1189-1200.
10. Graham WJ, B.J., Bullough CHW, *Can skilled attendance at delivery reduce maternal mortality in developing countries?* Safe Motherhood strategies: a review of the evidence 2001. **17**: p. 97-130.
11. Souza, J.P., et al., *Moving beyond essential interventions for reduction of maternal mortality (the WHO Multicountry Survey on Maternal and Newborn Health): a cross-sectional study.* Lancet, 2013. **381**(9879): p. 1747-1755.
12. World Health Organization, *Making pregnancy safer: the critical role of the skilled attendant: a joint statement by WHO, ICM and FIGO.* 2004.
13. Das, J.K., et al., *Evidence from facility level inputs to improve quality of care for maternal and newborn health: interventions and findings.* Reprod Health, 2014. **11**(2): p. S4.
14. Lassi, Z.S. and Z.A. Bhutta, *Community-based intervention packages for reducing maternal and neonatal morbidity and mortality and improving neonatal outcomes.* Cochrane Database Syst Rev, 2015(3).
15. Peabody, J.W., et al., *Comparison of vignettes, standardized patients, and chart abstraction: a prospective validation study of 3 methods for measuring quality.* JAMA, 2000. **283**(13): p. 1715-1722.
16. Peabody, J.W., et al., *Measuring the quality of physician practice by using clinical vignettes: a prospective validation study.* Ann Intern Med, 2004. **141**(10): p. 771-780.
17. Landry, E., et al., *Assessing the quality of record keeping for cesarean deliveries: results from a multicenter retrospective record review in five low-income countries.* BMC Pregnancy Childbirth, 2014. **14**(1): p. 139.

For peer review only - http://bmjopen.bmj.com/site/about/guidelines.xhtml

18. Wilunda, C., et al., *Availability, utilisation and quality of maternal and neonatal health care services in Karamoja region, Uganda: a health facility-based survey.* Reprod Health, 2015. **12**(1): p. 30.
19. Say, L., R.C. Pattinson, and A.M. Gülmezoglu, *WHO systematic review of maternal morbidity and mortality: the prevalence of severe acute maternal morbidity (near miss).* Reprod Health, 2004. **1**(1): p. 3.
20. Harvey, S.A., et al., *Are skilled birth attendants really skilled? A measurement method, some disturbing results and a potential way forward.* Bull World Health Organ, 2007. **85**: p. 783-790.
21. Lohela, T.J., et al., *Competence of health workers in emergency obstetric care: an assessment using clinical vignettes in Brong Ahafo region, Ghana.* BMJ Open, 2016. **6**(6): p. e010963.
22. Vesel, L., et al., *Quality of newborn care: a health facility assessment in rural Ghana using survey, vignette and surveillance data.* BMJ Open, 2013. **3**(5): p. e002326.
23. World Health Organization., *Integrated management of pregnancy and childbirth: WHO recommended interventions for improving maternal and newborn health*. 2009: World Health Organization.
24. Ministry of Health and Family Welfare (MOHFW), *Bangladesh Health Bulletin 2017*. 2018, Management Information System: Directorate General of Health Services: Bangladesh.
25. Nyamtema, A.S., D.P. Urassa, and J. van Roosmalen, *Maternal health interventions in resource limited countries: a systematic review of packages, impacts and factors for change.* BMC Pregnancy Childbirth, 2011. **11**(1): p. 30.
26. Islam, M.T., et al., *Implementation of emergency obstetric care training in Bangladesh: lessons learned.* Reprod Health Matters, 2006. **14**(27): p. 61-72.
27. Varghese, B., et al., *Limited effectiveness of a skills and drills intervention to improve emergency obstetric and newborn care in Karnataka, India: a proof-of-concept study.* Glob Health Sci Pract, 2016. **4**(4): p. 582-593.
28. Spencer, L.M., *Competency assessment methods.* What Works: Assessment, Development, and Measurement, 1997. **1**: p. 1-36.
29. Kak, N., B. Burkhalter, and M.-A. Cooper, *Measuring the competence of healthcare providers.* Operations Research Issue Paper, 2001. **2**(1): p. 1-28.
30. Utz, B., T. Kana, and N. Van Den Broek, *Practical aspects of setting up obstetric skills laboratories–a literature review and proposed model.* Midwifery, 2015. **31**(4): p. 400-408.
31. Huicho, L., et al., *How much does quality of child care vary between health workers with differing durations of training? An observational multicountry study.* Lancet, 2008. **372**(9642): p. 910-916.
32. Fulton, B.D., et al., *Health workforce skill mix and task shifting in low income countries: a review of recent evidence.* Hum Resour Health, 2011. **9**(1): p. 1.
33. Bogren, M.U., et al., *Towards a midwifery profession in Bangladesh–a systems approach for a complex world.* BMC Pregnancy Childbirth, 2015. **15**(1): p. 325.
34. UNFPA Bangladesh. *UNFPA's success in saving mothers and babies' lives in Bangladesh: 1,143 Midwives deployed to government health facilities*. 2018; Available from: https://bangladesh.unfpa.org/en/news/unfpas-success-saving-mothers-and-babies%E2%80%99-lives-bangladesh-1143-midwives-deployed-government.
35. Moxon, S.G., et al., *Service readiness for inpatient care of small and sick newborns: what do we need and what can we measure now?* J Glob Health, 2018. **8**(1).

For peer review only

For peer review only - http://bmjopen.bmj.com/site/about/guidelines.xhtml

36. Jayanna, K., et al., *Assessment of facility readiness and provider preparedness for dealing with postpartum haemorrhage and pre-eclampsia/eclampsia in public and private health facilities of northern Karnataka, India: a cross-sectional study.* BMC Pregnancy Childbirth, 2014. **14**(1): p. 304.
37. While, A.E., *Competence versus performance: which is more important?* J Adv Nurs, 1994. **20**(3): p. 525-531.

For peer review only

For peer review only - http://bmjopen.bmj.com/site/about/guidelines.xhtml

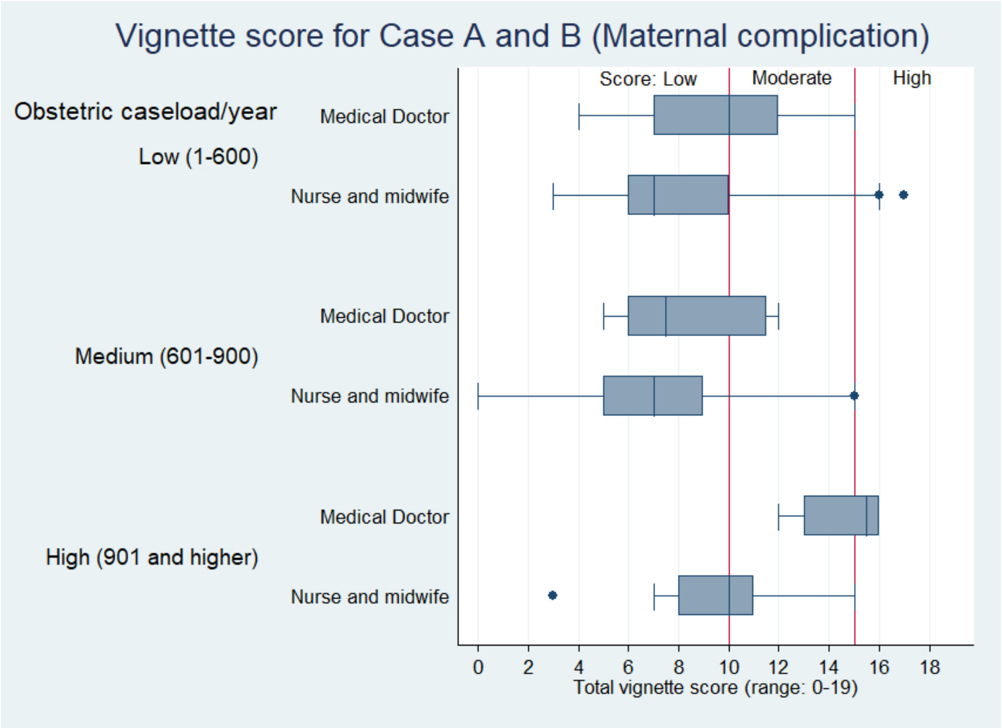

The vignette scores for obstetric complications by providers cadre and type of facility

72x52mm (300 x 300 DPI)

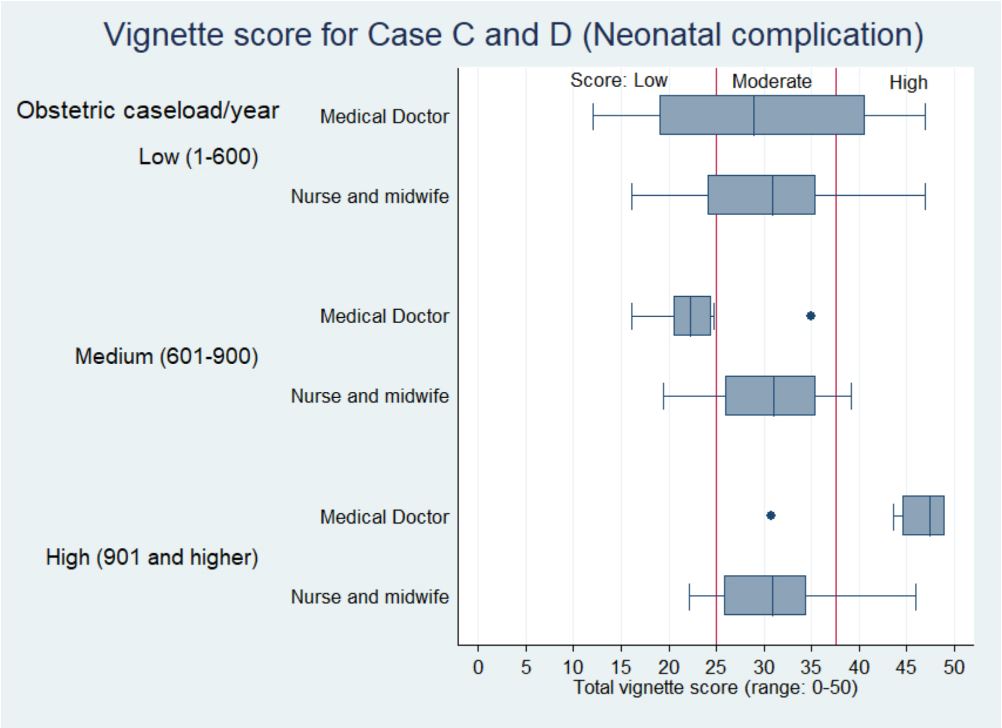

The vignette scores for neonatal complications by providers cadre and type of facility

72x52mm (300 x 300 DPI)

S1 Appendix: Obstetric and neonatal vignette scenarios

Case A (Eclampsia): Total score: 09

A 26-year-old woman who is 7 months pregnant comes in complaining of headaches, blurred vision and epigastric pain and her face looks swollen. In this facility, what would you usually do to establish a diagnosis?

1. Measure woman's blood pressure *+1 point if mentioned*
2. Check urine for protein *+1 point if mentioned*
3. Check her reflexes *+1 point if mentioned*
4. Check fetal heart rate *+1 point if mentioned*

Upon examination she had a blood pressure of 170/120, 3+ protein in her urine and brisk reflexes. How would she be managed at this facility?

1. Giving antihypertensive drug, e.g. hydralazine, labetalol, nifedipine *+1 point if mentioned*
2. Give Magnesium sulfate or, if not available, diazepam *+1 point if mentioned*
3. Give diuretics *+1 point if mentioned*
4. Have somebody stay with her all the time in case she starts having seizures *+1 point if mentioned*
5. Plan for delivery within the next 24 hours, or
6. Refer to other health facility immediately *+1 point if at least one out of two mentioned*

Case B (Antepartum haemorrhage): Total score: 10

A 35-year old woman who is 8 months pregnant comes to this facility because she has started to bleed heavily vaginally. She has no contractions and does not complain of any pain. In this facility, what would you usually do to establish a diagnosis?

1. Check woman's vital signs *+1 point if mentioned*
2. Check fetal heart rate *+1 point if mentioned*
3. Perform abdominal examination *+1 point if mentioned*
4. Will not perform vaginal examination *+1 point if mentioned*

The woman has a feeble pulse at 120/min, her systolic blood pressure is 85 and she is pale, sweating and breathing rapidly at 30 breaths per minute. Foetal heart sound is normal. There is no tenderness on abdominal examination. She is still bleeding vaginally, bright red blood.

For peer review only

You suspect placenta praevia and therefore do not perform a vaginal examination. How would such a patient be managed now?

1. Elevate legs to increase return of blood to the heart *+1 point if mentioned*
2. Give IV fluids rapidly *+1 point if mentioned*
3. Give oxygen by mask or nasal cannula *+1 point if mentioned*
4. Do ultrasound to confirm diagnosis *+1 point if mentioned*
5. Prepare for Caesarian section or refer to hospital where caesarean section can be done *+1 point if mentioned*
6. Give blood transfusion *+1 point if mentioned*

Case C (Care for very low birth weight baby): Total score: 20

A 17-year-old woman pregnant for 8 months delivered a baby at home. The baby was weighed and found to be 1.4kg. The baby was referred to your facility and suppose you had admitted her for immediate care. What would you do for this baby?

1. Detain for thorough examination
2. Ensure breastfeeding is established and provide support if necessary
3. Put the baby in an incubator
4. Teach the mother to keep baby Skin-to-skin or KMC
5. Check cord dressing and other potential sources of infection
6. Encourage and ensure hygiene in care
7. Refer to a hospital/another facility

Mother says the baby is not breastfeeding and was contemplating giving glucose solution. What would you do?

1. Watch her breastfeed her baby and teach her good positioning and attachment
2. Examine the baby's mouth to ensure there are no anatomical deformities
3. If baby not breast feeding, teach her to express the milk and feed with a clean cup
4. Encourage infant formula only if EBM is not possible and mother can afford
5. Educate her and encourage her to practice exclusive breastfeeding for 1st 6 months of the baby's life

Case D (Essential newborn care and resuscitation): Total score: 30

A woman presented here in labour. The FHR is more than 160bpm. On examination, her cervix was fully dilated, and the baby had the head in the perineum. The baby is delivered and is normal weight but did not cry after delivery; what would you do for this baby?

For peer review only

1. Dry quickly *score: 2.66*
2. Examine and suction mouth *score: 2.16*
3. Ensure extra warmth for the baby *score: 1.50*
4. Use bag and mask to ventilate if baby does not cry after suctioning *score: 2.53*
5. Apply cardiac massage if ventilation alone does not help *score: 1.16*

Suppose the resuscitation was successful, what would you do next?

1. Initiate breastfeeding immediately *score: 3.31*
2. skin-to-skin contact with mother *score: 4.34*
3. Ensure and encourage hygiene *score: 2.34*

During routine checking on the baby after about 2 hrs, you saw the baby sleeping alone and the mother is sleeping but not in touch with baby. There was no covering on the baby since it wriggled out of the mother's cloth. What would you do?

1. Feel if baby is too cold *score: 1.28*
2. Take the temperature with a thermometer *score: 1.53*
3. Give skin-to-skin care/kangaroo mother care by mother or put in incubator for rewarming *score: 3.94*
4. Check the ward to see if windows are open and close them all as well as any fans on the ward to prevent draught *score: 1. 41*
5. Let the mother breastfeed the baby immediately *score: 1.84*

For peer review only

Check-list: Revised Standards for Quality Improvement Reporting Excellence (SQUIRE 2.0)

September 15, 2015

Text Section and Item Name	Section or Item Description	Page number
1. Title	Indicate that the manuscript concerns an initiative to improve healthcare (broadly defined to include the quality, safety, effectiveness, patient-centeredness, timeliness, cost, efficiency, and equity of healthcare)	1
2. Abstract	a. Provide adequate information to aid in searching and indexing	2
	b. Summarize all key information from various sections of the text using the abstract format of the intended publication or a structured summary such as: background, local problem, methods, interventions, results, conclusions	2
**Introduction**	**Why did you start?**	
3. Problem description	Nature and significance of the local problem	4
4. Available knowledge	Summary of what is currently known about the problem, including relevant previous studies	4
5. Rationale	Informal or formal frameworks, models, concepts, and/or theories used to explain the problem, any reasons or assumptions that were used to develop the intervention(s), and reasons why the intervention(s) was expected to work	4
6. Specific aims	Purpose of the project and of this report	4-5
**Methods**	**What did you do?**	
7. Context	Contextual elements considered important at the outset of introducing the intervention(s)	5

For peer review only

8. Intervention(s)	a. Description of the intervention(s) in sufficient detail that others could reproduce it	5
	b. Specifics of the team involved in the work	6
9. Study of the Intervention(s)	a. Approach chosen for assessing the impact of the intervention(s)	5-6
	b. Approach used to establish whether the observed outcomes were due to the intervention(s)	5-6
10. Measures	a. Measures chosen for studying processes and outcomes of the intervention(s), including rationale for choosing them, their operational definitions, and their validity and reliability	5-6
	b. Description of the approach to the ongoing assessment of contextual elements that contributed to the success, failure, efficiency, and cost	5-6
	c. Methods employed for assessing completeness and accuracy of data	6
11. Analysis	a. Qualitative and quantitative methods used to draw inferences from the data	6-7
	b. Methods for understanding variation within the data, including the effects of time as a variable	Not applicable
12. Ethical Considerations	Ethical aspects of implementing and studying the intervention(s) and how they were addressed, including, but not limited to, formal ethics review and potential conflict(s) of interest	7
**Results**	**What did you find?**	
13. Results	a. Initial steps of the intervention(s) and their evolution over time (e.g., time-line diagram, flow chart, or table), including modifications made to the intervention during the project	7-9

For peer review only

	b. Details of the process measures and outcome	10-12
	c. Contextual elements that interacted with the intervention(s)	7-9
	d. Observed associations between outcomes, interventions, and relevant contextual elements	11-12
	e. Unintended consequences such as unexpected benefits, problems, failures, or costs associated with the intervention(s).	Not applicable
	f. Details about missing data	Not applicable
**Discussion**	**What does it mean?**	
14. Summary	a. Key findings, including relevance to the rationale and specific aims	13
	b. Particular strengths of the project	13
15. Interpretation	a. Nature of the association between the intervention(s) and the outcomes	13-14
	b. Comparison of results with findings from other publications	13-14
	c. Impact of the project on people and systems	13-14
	d. Reasons for any differences between observed and anticipated outcomes, including the influence of context	13-14
	e. Costs and strategic trade-offs, including opportunity costs	Not applicable
16. Limitations	a. Limits to the generalizability of the work	15
	b. Factors that might have limited internal validity such as confounding, bias, or imprecision in the design, methods, measurement, or analysis	15
	c. Efforts made to minimize and adjust for limitations	15
17. Conclusions	a. Usefulness of the work	15-16

For peer review only

	b. Sustainability	16
	c. Potential for spread to other contexts	16
	d. Implications for practice and for further study in the field	16
	e. Suggested next steps	16
**Other information**		
18. Funding	Sources of funding that supported this work. Role, if any, of the funding organization in the design, implementation, interpretation, and reporting	16

For peer review only
